# Sparse Estimation Strategies in Linear Mixed Effect Models for High-Dimensional Data Application

**DOI:** 10.3390/e23101348

**Published:** 2021-10-15

**Authors:** Eugene A. Opoku, Syed Ejaz Ahmed, Farouk S. Nathoo

**Affiliations:** 1 Department of Mathematics and Statistics, University of Victoria, Victoria, BC V8P 5C2, Canada; nathoo@uvic.ca; 2 Department of Mathematics and Statistics, Brock University, St. Catharines, ON L2S 3A1, Canada; sahmed5@brocku.ca

**Keywords:** linear mixed model, ridge estimation, pretest and shrinkage estimation, multicollinearity, asymptotic bias and risk, LASSO estimation, high-dimensional data

## Abstract

In a host of business applications, biomedical and epidemiological studies, the problem of multicollinearity among predictor variables is a frequent issue in longitudinal data analysis for linear mixed models (LMM). We consider an efficient estimation strategy for high-dimensional data application, where the dimensions of the parameters are larger than the number of observations. In this paper, we are interested in estimating the fixed effects parameters of the LMM when it is assumed that some prior information is available in the form of linear restrictions on the parameters. We propose the pretest and shrinkage estimation strategies using the ridge full model as the base estimator. We establish the asymptotic distributional bias and risks of the suggested estimators and investigate their relative performance with respect to the ridge full model estimator. Furthermore, we compare the numerical performance of the LASSO-type estimators with the pretest and shrinkage ridge estimators. The methodology is investigated using simulation studies and then demonstrated on an application exploring how effective brain connectivity in the default mode network (DMN) may be related to genetics within the context of Alzheimer’s disease.

## 1. Introduction

In many fields such as bio-informatics, physical biology, and epidemiology, the response of interest is represented by repeated measures of some variables of interest that are collected over a specified time period for different independent subjects or individuals. These types of data are commonly encountered in medical research where the responses are subject to various time-dependent and time-constant effects such as pre- and post-treatment types, gender effect, and baseline measures, among others. A widely-used statistical tool in the analysis and modeling of longitudinal and repeated measures data is the linear mixed effects model (LMM) [1,2]. This model provides an effective and flexible way to describe the means and the covariance structures of a response variable after accounting for within subject correlation.

The rapid growth in the size and scope of longitudinal data has created a need for innovative statistical strategies in longitudinal data analysis. Classical methods are based on the assumption that the number of predictors is less than the number of observations. However, there is an increasing demand for efficient prediction strategies for analysis of high-dimensional data, where the number of observed data elements (sample size) are smaller than the number of predictors in a linear model context. Existing techniques that deal with high-dimensional data mostly rely on various penalized estimators. Due to the trade-off between model complexity and model prediction, the statistical inference of model selection becomes an extremely important and challenging problem in high-dimensional data analysis.

Over the years, many penalized regularization approaches have been developed to do variable selection and estimation simultaneously. Among them, the least absolute shrinkage and selection operator (LASSO) is commonly used [3]. It is a useful estimation technique in part due to its convexity and computational efficiency. The LASSO approach is based on an $\ell_{1}$ penalty for regularization of regression parameters. Ref. [4] provides a comprehensive summary of the consistency properties of the LASSO approach. Related penalized likelihood methods have been extensively studied in the literature, see for example [5,6,7,8,9,10]. The penalized likelihood methods have a close connection to Bayesian procedures. Thus, the LASSO estimate corresponds to a Bayes method that puts a Laplacian (double-exponential) prior on the regression coefficients [11,12].

In this paper, our interest lies in estimating the fixed effect parameters of the LMM using a ridge estimation technique when it is assumed that some prior information is available in the form of potential linear restrictions on the parameters. One possible source of prior information is using a Bayesian approach. An alternative source of prior information may be obtained from previous studies or expert knowledge that search for or assume sparsity patterns.

We consider the problem of fixed effect parameter estimation for LMMs when there exist many predictors relative to the sample size. These predictors may be classified into two groups: sparse and non-sparse. Thus, there are two choices to be considered: a full model with all predictors, and a sub-model that contains only non-sparse predictors. When the sub-model based on available subspace information is true (i.e., the assumed restriction holds), it then provides more efficient statistical inferences than those based on a full model. In contrast, if the sub-model is not true, the estimates could become biased and inefficient. The consequences of incorporating subspace information therefore depend on the quality or reliability of the information being incorporated into the estimation procedure. One way to deal with uncertain subspace information is to use a pretest estimation strategy. The validity of the information is tested before incorporation into a final estimator. Another approach is shrinkage estimation, which shrinks the full model estimator to the sub-model estimator by utilizing subspace information. Besides these estimation strategies, there is a growing literature on simultaneous model selection and estimation. These approaches are known as penalty strategies. By shrinking some regression coefficients toward zero, the penalty methods simultaneously select a sub-model and estimate its regression parameters. Several authors have investigated the pretest, shrinkage, and penalty estimation strategies in partial linear model, Poisson regression model, and Weibull censored regression model [13,14,15].

To formulate the problem, we suppose that the vector of the fixed effects parameter β in the LMM can be partitioned into two sub-vectors $\beta ={\left(\beta_{1}^{\prime },\beta_{2}^{\prime }\right)}^{\prime }$, where $\beta_{1}$ is the coefficient vector of non-sparse predictors and $\beta_{2}$ is the coefficient vector of sparse predictors. Our interest lies in the estimation of $\beta_{1}$ when $\beta_{2}$ is close to zero. To deal with this problem in the context of low dimensional data, ref. [16] propose an improved estimation strategy using sub-model selection and post-estimation for the LMM. Within this framework, linear shrinkage and shrinkage pretest estimation strategies are developed, which combine full model and sub-model estimators in an effective way as a trade-off between bias and variance. Ref. [17] extend this study by using a likelihood ratio test to develop James–Stein shrinkage and pretest estimation methods based on LMM for longitudinal data. In addition, the non-penalty estimators are compared with several penalty estimators (LASSO, adaptive LASSO and Elastic Net) for best performance.

In most real data situations, there is also the problem of multicollinearity among predictor variables for high-dimensional data. Various biased estimation techniques such as shrinkage estimation, partial least squares estimation [18] and Liu estimators [19] have been implemented to deal with this problem, but the widely used technique is ridge estimation [20]. The ridge estimator overcomes the weakness of the least squares estimator with a smaller mean squared error. To overcome and combat multicollinearity, ref. [21] propose pretest and Stein-type ridge regression estimators for linear and partially linear models. Furthermore, ref. [22] also develop shrinkage estimation based on Liu regression to overcome multicollinearity in linear models.

Our primary focus is on the estimation and prediction problem for linear mixed effect models when there are many potential predictors that have a weak or no influence on the response of interest. This method simultaneously controls overfitting using general least square estimation with a roughness penalty. We propose pretest and shrinkage estimation strategies using the ridge estimation technique as a base estimator and numerically compare their performance with the LASSO and adaptive LASSO estimators. Our proposed estimation strategy is applied to both high-dimensional and low-dimensional data.

The rest of this article is organized as follows. In Section 2, we present the linear mixed effect model and the proposed estimation techniques. We introduce the full and sub-model estimators based on ridge estimation. Thereafter, we construct the pretest and shrinkage ridge estimators. Section 3 provides the asymptotic bias and risk of these estimators. A Monte Carlo simulation is used to evaluate the performance of the estimators including a comparison with the lasso-type estimators, and the results are reported in Section 4. Section 5 presents a demonstration of the proposed methodology on a high-dimensional resting-state effective brain connectivity and genetic data. We also illustrate the proposed estimation methods in an application to a low-dimensional Amsterdam growth and health study. Section 6 presents a discussion with recommendations.

## 2. Model and Estimation Strategies

In this section, we present the linear mixed effect model and the proposed estimation strategies.

### 2.1. Linear Mixed Model

Suppose that we have a sample of *N* subjects. For the $i^{th}$ subject, we collect the response variable $y_{ij}$ for the jth time, where $i=1\ldots ,n;j=1\ldots ,n_{i}$ and $N=\sum_{i=1}^{n}n_{i}$. Let $Y_{i}={\left(y_{i1},\ldots y_{in_{i}}\right)}^{\prime }$ denotes the $n_{i}\times 1$ vector of responses from the ith subject. Let $X_{i}={\left(x_{i1},\ldots ,x_{in_{i}}\right)}^{\prime }$ and $Z_{i}={\left(z_{i1},\ldots ,z_{in_{i}}\right)}^{\prime }$ be $n_{i}\times$ p and $n_{i}\times$ q known fixed-effects and random-effect design matrix for the ith subject of full rank *p* and *q*, respectively. The linear mixed effect model [1] for a vector of repeated responses $Y_{i}$ on the ith subject is assumed to have the form

(1) $$Y_{i}=X_{i}\beta +Z_{i}a_{i}+\epsilon_{i},$$

where $\beta ={\left(\beta_{1},\ldots ,\beta_{p}\right)}^{\prime }$ is the p × 1 vector of unknown fixed-effect parameters or regression coefficients, $a_{i}$ is the q × 1 vector of unobservable random effects for the ith subject, assumed to come from a multivariate normal distribution with zero mean and a covariance matrix **G**, where **G** is an unknown q×q covariance matrix and $\epsilon_{i}$ denotes $n_{i}\times$ 1 vector of error terms assumed to be normally distributed with zero mean, covariance matrix $\sigma^{2}I_{n_{i}}$. Further, $\epsilon_{i}$ are assumed to be independent of the random effects $a_{i}$.

The marginal distribution for the response $y_{i}$ is normal with mean $X_{i}\beta$ and covariance matrix $Cov\left(Y_{i}\right)=Z_{i}\sigma_{i}^{2}Z_{i}^{T}+\sigma^{2}I_{n}.$ By stacking the vectors, the mixed model can be can be expressed as Y=Xβ+Za+ϵ. From the Equation (1), the distribution of the model follows $Y∼N_{n}\left(X\beta ,V\right)$, where E(Y)=Xβ with covariance, $V=\sum_{i=1}^{n}Z_{i}\sigma_{i}^{2}Z_{i}^{T}+\sigma^{2}I_{n}$.

### 2.2. Ridge Full Model and Sub-Model Estimator

The generalized least square estimator (GLS) is defined as ${\hat{\beta }}^{\mathrm{GLS}}={\left(X^{T}V^{-1}X\right)}^{-1}X^{T}V^{-1}Y$ and the ridge full model estimator can be obtained by introducing a penalized regression so that $\hat{\beta }=\arg\min_{\beta }\left\{{\left(Y-X\beta \right)}^{T}V^{-1}\left(Y-X\beta \right)+k\beta^{T}\beta \right\}$ and ${\hat{\beta }}^{\mathrm{Ridge}}={\left(X^{T}V^{-1}X+kI\right)}^{-1}X^{T}V^{-1}Y$, where ${\hat{\beta }}^{\mathrm{Ridge}}$ is the ridge full model estimator and k∈[0,∞) is the tuning parameter. If k = 0, ${\hat{\beta }}^{\mathrm{Ridge}}$ is the GLS estimator and ${\hat{\beta }}^{\mathrm{Ridge}}=0$ for *k* is sufficiently large. We select the value of *k* using cross validation.

We let $X=(X_{1},X_{2})$, where $X_{1}$ is an $n\times p_{1}$ sub-matrix containing the non-sparse predictors and $X_{2}$ is an $n\times p_{2}$ sub-matrix that contains the sparse predictors. Accordingly, $\beta =(\beta_{1},\beta_{2})$ where $\beta_{1}$ and $\beta_{2}$ have dimensions $p_{1}$ and $p_{2}$, respectively, with $p_{1}+p_{2}=p$, $p_{i}\geq 0$ for *i* = 1, 2.

A sub-model is defined as $Y=X\beta +\mathrm{Za}+\epsilon \;\mathrm{subject}\;\mathrm{to}\;\beta^{T}\beta \leq \phi \;\mathrm{and}\;\beta_{2}=0$ which corresponds to $Y=X_{1}\beta_{1}+\mathrm{Za}+\epsilon \;\mathrm{subject}\;\mathrm{to}\;\beta_{1}^{T}\beta_{1}\leq \phi .$ The sub-model estimator ${\hat{\beta }}_{1}^{\mathrm{RSM}}$ of $\beta_{1}$ has the form ${\hat{\beta }}_{1}^{\mathrm{RSM}}={\left(X_{1}^{T}V^{-1}X_{1}+kI\right)}^{-1}X_{1}^{T}V^{-1}Y.$ We denote ${\hat{\beta }}_{1}^{\mathrm{RFM}}$ as the full model ridge estimator of $\beta_{1}$ and given as ${\hat{\beta }}_{1}^{\mathrm{RFM}}={\left(X_{1}^{T}V^{-1/2}M_{X_{2}}V^{-1/2}X_{1}+kI\right)}^{-1}X_{1}^{T}V^{-1/2}M_{X_{2}}V^{-1/2}Y,$ where $M_{X_{2}}=I-P=I-V^{-1/2}X_{2}{\left(X_{2}V^{-1}X_{2}\right)}^{-1}X_{2}^{T}V^{-1/2}.$

### 2.3. Pretest Ridge Estimation Strategy

Generally, the sub-model estimator will be more efficient than the full model estimator if the information embodied in the imposed linear restrictions is valid, thus $\beta_{2}$ is close to zero. However, if the information is not valid the sub-model estimator is likely to be more biased and may have a higher risk than the full model estimator. There is, therefore, some doubt as to whether or not to impose the restrictions on the model’s parameter. It is in response to this uncertainty that a statistical test may be used to determine the validity of the proposed restrictions. Accordingly, the procedure to follow in practice is pretest the validity of the restrictions and if the outcome of the pretest suggests that they are correct then the model parameters are estimated incorporating the restrictions. If the pretest rejects the restrictions then the parameters are estimated from the sample information alone. This motivates the consideration of the pretest estimation strategy for the LMM.

The pretest estimator is a combination of the full model estimator ${\hat{\beta }}_{1}^{\mathrm{RFM}},$ and sub-model estimator ${\hat{\beta }}_{1}^{\mathrm{RSM}},$ through an indicator function $I(L_{n}\leq d_{n,\alpha })$, where $L_{n}$ is an appropriate test statistic to test $H_{0}:\beta_{2}=0$ versus $H_{A}:\beta_{2}\neq 0$. Moreover, $d_{n,\alpha }$ is an α level critical value based on distribution of $L_{n}$ under $H_{0}$. We define test statistics based on the log-likelihood ratio test as $L_{n}=2\left\{\ell^{*}\left({\hat{\beta }}^{\mathrm{RFM}}|Y\right)-\ell^{*}\left({\hat{\beta }}^{\mathrm{RSM}}|Y\right)\right\}$.

Under $H_{0}$, the test statistic $L_{n}$ follows asymptotic chi-square distribution with $p_{2}$ degrees of freedom. The pretest test ridge estimator ${\hat{\beta }}_{1}^{\mathrm{RPT}}$ of $\beta_{1}$ is then defined by

$${\hat{\beta }}_{1}^{\mathrm{RPT}}={\hat{\beta }}_{1}^{\mathrm{RFM}}-\left({\hat{\beta }}_{1}^{\mathrm{RFM}}-{\hat{\beta }}_{1}^{\mathrm{RSM}}\right)I\left(L_{n}\leq d_{n,\alpha }\right),\;p_{2}\geq 1$$.

### 2.4. Shrinkage Ridge Estimation Strategy

The pre-test estimator is a discontinuous function of the sub-model ${\hat{\beta }}_{1}^{\mathrm{RSM}}$ and full model ${\hat{\beta }}_{1}^{\mathrm{RFM}}$, which depends on the hard threshold $\left(d_{n,\alpha }=\chi_{p_{2},\alpha }^{2}\right)$. We address this limitation by defining the shrinkage ridge estimator based on soft thresholding. The shrinkage ridge estimator (RSE) of $\beta_{1}$, denoted as ${\hat{\beta }}_{1}^{\mathrm{RSE}},$ is defined as

$${\hat{\beta }}_{1}^{\mathrm{RSE}}={\hat{\beta }}_{1}^{\mathrm{RSM}}+\left({\hat{\beta }}_{1}^{\mathrm{RFM}}-{\hat{\beta }}_{1}^{\mathrm{RSM}}\right)\left(1-\left(p_{2}-2\right)L_{n}^{-1}\right),\;p_{2}\geq 3.$$

Here, ${\hat{\beta }}_{1}^{\mathrm{RSE}}$ is the linear combination of the full model ${\hat{\beta }}_{1}^{\mathrm{RFM}}$ and sub-model ${\hat{\beta }}_{1}^{\mathrm{RSM}}$ estimates. If $L_{n}\leq \left(p_{2}-2\right),$ then a relatively large weight is placed on ${\hat{\beta }}_{1}^{\mathrm{RSM}}$ otherwise, more weight is on ${\hat{\beta }}_{1}^{\mathrm{RFM}}$. A setback with ${\hat{\beta }}_{1}^{\mathrm{RSE}}$ is that it is not a convex combination of ${\hat{\beta }}_{1}^{\mathrm{RFM}}$ and ${\hat{\beta }}_{1}^{\mathrm{RSM}}$. This can cause over-shrinkage, which gives the estimator opposite sign of ${\hat{\beta }}_{1}^{\mathrm{RFM}}$. This could happen if $\left(p_{2}-2\right)L_{n}^{-1}$ is larger than one. To counter this, we use the positive-part shrinkage ridge estimator (RPS) defined as

$${\hat{\beta }}_{1}^{\mathrm{RPS}}={\hat{\beta }}_{1}^{\mathrm{RSM}}+\left({\hat{\beta }}_{1}^{\mathrm{RFM}}-{\hat{\beta }}_{1}^{\mathrm{RSM}}\right){\left(1-\left(p_{2}-2\right)L_{n}^{-1}\right)}^{+},p_{2}\geq 3$$

where ${\left(1-\left(p_{2}-2\right)L_{n}^{-1}\right)}^{+}=\max\left(0,1-\left(p_{2}-2\right)L_{n}^{-1}\right).$ The RPS estimator will control possible over-shrinking in the RSE estimator.

## 3. Asymptotic Results

In this section, we derive the asymptotic distributional bias and risk of the estimators considered in Section 2. We examine the properties of the estimators for increasing *n* and as $\beta_{2}$ approaches the null vector under the sequence of local alternatives defined as

(2) $$K_{n}:\beta_{2}=\beta_{2(n)}=\frac{\kappa }{\sqrt{n}},$$

where $\kappa ={\left(\kappa_{1},\kappa_{2}\ldots ,\kappa_{p_{2}}\right)}^{\prime }\in R^{p_{2}}$ is a fixed vector. The vector $\frac{\kappa }{\sqrt{n}}$ is a measure of how far local alternatives $K_{n}$ differ from the subspace information $\beta_{2}=0$. In order to evaluate the performance of the estimators, we define the asymptotic distributional bias of the estimator ${\hat{\beta }}_{1}^{*}$ as

$$\mathrm{ADB}\left({\hat{\beta }}_{1}^{*}\right)=\lim_{n\to \infty }E\left\{\sqrt{n}\left({\hat{\beta }}_{1}^{*}-\beta_{1}\right)\right\},$$

In order to compute the risk functions, we first compute the asymptotic covariance of the estimators. The asymptotic covariance of an estimator ${\hat{\beta }}_{1}^{*}$ is expressed as

$$\mathrm{Cov}\left({\hat{\beta }}_{1}^{*}\right)=\lim_{n\to \infty }E\left\{n\left({\hat{\beta }}_{1}^{*}-\beta_{1}\right){\left({\hat{\beta }}_{1}^{*}-\beta_{1}\right)}^{T}\right\}.$$

Following the asymptotic covariance matrix, we define the asymptotic risk of an estimator ${\hat{\beta }}_{1}^{*}$ as $R\left({\hat{\beta }}_{1}^{*}\right)=\mathrm{tr}\left(Q\mathrm{Cov}({\hat{\beta }}_{1}^{*})\right)$. **Q** is a positive definite matrix of weights with dimensions of p×p. We set **Q** = **I** in this study.

**Assumption** **1.** *We make the following two regularity conditions to establish the asymptotic properties of the estimators.*

*1.*$\frac{1}{n}\max_{1\leq i\leq n}^{}x_{i}^{T}{\left[X^{T}V^{-1}X\right]}^{-1}x_{i}\to$***0**as n*→∞,*where*$x_{i}^{T}$*is the ith row of**X***.

*2.*$B_{n}=n^{-1}{\left[X^{T}V^{-1}X\right]}^{-1}\to B,$*for some finite**B*** = $\left(\begin{array}{cc} B_{11} & B_{12}\\ B_{21} & B_{22} \end{array}\right)$.

**Theorem** **1.** *For*k<∞, *If*$k/\sqrt{n}\to \lambda_{o}$*and **B** is non-singular, the distribution of the full model ridge estimator*, ${\hat{\beta }}_{n}^{\mathrm{RFM}}$*is*

$$\sqrt{n}\left({\hat{\beta }}_{n}^{\mathrm{RFM}}-\beta \right)\overset{D}{\to }N\left(-\lambda_{o}B^{-1}\beta ,B^{-1}\right),$$

*where*$\overset{D}{\to }$*denotes convergence in distribution.*

**Proof.**  See Theorem 2 in [23]. □

**Proposition** **1.** *Assuming the above assumption 1 together with Theorem 1 hold, under the local alternatives*$K_{n}$, *we have*

$$\left(\begin{array}{c} \varphi_{1}\\ \varphi_{3} \end{array}\right)\overset{D}{\to }N\left[\left(\begin{array}{c} -\mu_{11.2}\\ \delta \end{array}\right),\left(\begin{array}{cc} B_{11.2}^{-1} & \Phi \\ \Phi & \Phi \end{array}\right)\right],$$

$$\left(\begin{array}{c} \varphi_{3}\\ \varphi_{2} \end{array}\right)\overset{D}{\to }N\left[\left(\begin{array}{c} \delta \\ -\gamma \end{array}\right),\left(\begin{array}{cc} \Phi & 0\\ 0 & B_{11}^{-1} \end{array}\right)\right],$$

where $\varphi_{1}=\sqrt{n}\left({\hat{\beta }}_{1}^{\mathrm{RFM}}-\beta_{1}\right)$, $\varphi_{2}=\sqrt{n}\left({\hat{\beta }}_{1}^{\mathrm{RSM}}-\beta_{1}\right)$, $\varphi_{3}=\sqrt{n}\left({\hat{\beta }}_{1}^{\mathrm{RFM}}-{\hat{\beta }}_{1}^{\mathrm{RSM}}\right)$, $\gamma =\mu_{11.2}+\delta$, $\delta =B_{11}^{-1}B_{12}\kappa$, $\Phi =B_{11}^{-1}B_{12}B_{22.1}^{-1}B_{21}B_{11}^{-1}$, $B_{22.1}=B_{22}-B_{21}B_{11}^{-1}B_{12}$, $\mu =-\lambda_{o}B^{-1}\beta =\left(\begin{array}{c} \mu_{1}\\ \mu_{2} \end{array}\right)$ and $\mu_{11.2}=\mu_{1}-B_{12}B_{22}^{-1}\left(\left(\beta_{2}-\kappa \right)-\mu_{2}\right)$.

**Proof.**  See Appendix A. □

**Theorem** **2.** *Under the condition of Theorem 1 and the local alternatives*$K_{n}$, *the ADBs of the proposed estimators are*

$$\begin{array}{cc} ADB({\hat{\beta }}_{1}^{\mathrm{RFM}}) & =-\mu_{11.2},\\ ADB({\hat{\beta }}_{1}^{\mathrm{RSM}}) & =-\mu_{11.2}-B_{11}^{-1}B_{12}\delta =-\gamma ,\\ ADB({\hat{\beta }}_{1}^{\mathrm{RPT}}) & =-\mu_{11.2}-\delta H_{p_{2}+2}\left(\chi_{p_{2},\alpha }^{2};\Delta \right),\\ ADB({\hat{\beta }}_{1}^{\mathrm{RSE}}) & =-\mu_{11.2}-\left(p_{2}-2\right)\delta E\left(\chi_{p_{2}+2}^{-2}\left(\Delta \right)\right),\\ ADB({\hat{\beta }}_{1}^{\mathrm{RPS}}) & =-\mu_{11.2}-\delta H_{p_{2}+2}\left(\chi_{p_{2}-2}^{2};\Delta \right)\}-\left(p_{2}-2\right)\delta E\left\{\chi_{p_{2}+2}^{-2}\left(\Delta \right)I\left(\chi_{p_{2}+2}^{-2}>p_{2}-2\right)\right\}, \end{array}$$

*where*$\Delta =\kappa^{T}B_{22.1}^{-1}\kappa$, $B_{22.1}=B_{22}-B_{21}B_{11}^{-1}B_{12}$, and $H_{v}\left(x;\Delta \right)$*is the cumulative distribution function of the non-central chi-squared distribution with non-centrality parameter* Δ *and v degrees of freedom, and* $E(\chi_{v}^{-2j}\left(\Delta \right))$ *is the expected value of the inverse of a non-central* $\chi^{2}$ *distribution with v degrees of freedom and non-centrality parameter* Δ,

$$E\left(\chi_{v}^{-2j}\left(\Delta \right)\right)=\int_{0}^{\infty }x^{-2j}dH_{v}\left(x,\Delta \right).$$

**Proof.**  See Appendix B.1. □

Since the ADBs of the estimators are in non-scalar form, we define the following asymptotic quadratic bias (AQDB) of ${\hat{\beta }}_{1}^{*}$ by

$$\mathrm{AQDB}\left({\hat{\beta }}_{1}^{*}\right)={\left(\mathrm{ADB}({\hat{\beta }}_{1}^{*})\right)}^{\prime }B_{11.2}\left(\mathrm{ADB}({\hat{\beta }}_{1}^{*})\right),$$

where $B_{11.2}=B_{11}-B_{12}B_{22}^{-1}B_{21}.$

**Corollary** **1.** *Suppose Theorem 2 holds. Then, under*$\left\{K_{n}\right\}$, *the AQDBs of the estimators are*

$$\begin{array}{cc} \mathrm{AQDB}({\hat{\beta }}_{1}^{\mathrm{RFM}}) & =\mu_{11.2}^{T}B_{11.2}\mu_{11.2},\\ \mathrm{AQDB}({\hat{\beta }}_{1}^{\mathrm{RSM}}) & =\gamma^{T}B_{11.2}\gamma ,\\ \mathrm{AQDB}({\hat{\beta }}_{1}^{\mathrm{RPT}}) & =\mu_{11.2}^{T}B_{11.2}\mu_{11.2}+\mu_{11.2}^{T}B_{11.2}\delta H_{p_{2}+2}\left(\chi_{p_{2}}^{2};\Delta \right)\\ & +\delta^{T}B_{11.2}\mu_{11.2}H_{p_{2}+2}\left(\chi_{p_{2}}^{2};\Delta \right)+\delta^{T}B_{11.2}\delta H_{p_{2}+2}^{2}\left(\chi_{p_{2}}^{2};\Delta \right),\\ \mathrm{AQDB}({\hat{\beta }}_{1}^{\mathrm{RSE}}) & =\mu_{11.2}^{T}B_{11.2}\mu_{11.2}+\left(p_{2}-2\right)\mu_{11.2}^{T}B_{11.2}\delta E\left(\chi_{p_{2}+2}^{-2}\left(\Delta \right)\right)\\ & +\left(p_{2}-2\right)\delta^{T}B_{11.2}\mu_{11.2}E\left(\chi_{p_{2}+2}^{-2}\left(\Delta \right)\right)+{\left(p_{2}-2\right)}^{2}\delta^{T}B_{11.2}\delta {\left(E\left(\chi_{p_{2}+2}^{-2}\left(\Delta \right)\right)\right)}^{2},\\ \mathrm{AQDB}({\hat{\beta }}_{1}^{\mathrm{RPS}}) & =\mu_{11.2}^{T}B_{11.2}\mu_{11.2}+\left(\delta^{T}B_{11.2}\mu_{11.2}+\mu_{11.2}^{T}B_{11.2}\delta \right)[H_{p_{2}+2}\left(p_{2}-2;\Delta \right)\\ & +\left(p_{2}-2\right)E\left\{\chi_{p_{2}+2}^{-2}\left(\Delta \right)I\left(\chi_{p_{2}+2}^{-2}\left(\Delta \right)>p_{2}-2\right)\right\}]+\delta^{T}B_{11.2}\delta [H_{p_{2}+2}\left(p_{2}-2;\Delta \right)\\ & +\left(p_{2}-2\right)E\left\{\chi_{p_{2}+2}^{-2}\left(\Delta \right)I\left(\chi_{p_{2}+2}^{-2}\left(\Delta \right)>p_{2}-2\right)\right\}{]}^{2}. \end{array}$$

When $B_{11.2}=0$, the AQDB of all estimators are equivalent, and the estimators are therefore asymptotically unbiased. If we assume that $B_{11.2}\neq 0$, the results for the bias of the estimators can be summarized as follows:

1. The AQDB of ${\hat{\beta }}_{1}^{\mathrm{RSM}}$ is an unbounded function of $\gamma^{T}B_{11.2}\gamma$.
2. The AQDB of ${\hat{\beta }}_{1}^{\mathrm{RPT}}$ starts from $\mu_{11.2}^{T}B_{11.2}\mu_{11.2}$ at Δ=0, and when Δ increases, it increases to the maximum and then decreases to zero.
3. The characteristics of ${\hat{\beta }}_{1}^{\mathrm{RSE}}$ and ${\hat{\beta }}_{1}^{\mathrm{RPS}}$ are similar to ${\hat{\beta }}_{1}^{\mathrm{RPT}}$. The AQDB of ${\hat{\beta }}_{1}^{\mathrm{RSE}}$ and ${\hat{\beta }}_{1}^{\mathrm{RPS}}$ similarly start from $\mu_{11.2}^{T}B_{11.2}\mu_{11.2}$ at Δ=0, and increase to a point, and then decrease towards zero, since $E\left\{\chi_{p_{2}+2}^{-2}\left(\Delta \right)\right\}$ is a non-increasing on of Δ.

**Theorem** **3.** *Suppose Theorem 1 holds and under the local alternatives*$K_{n}$, *the covariance matrices of the estimators are*

$$\begin{array}{cc} Cov({\hat{\beta }}_{1}^{\mathrm{RFM}}) & =B_{11.2}^{-1}+\mu_{11.2}\mu_{11.2}^{T},\\ Cov({\hat{\beta }}_{1}^{\mathrm{RSM}}) & =B_{11}^{-1}+\gamma \gamma^{T},\\ Cov({\hat{\beta }}_{1}^{\mathrm{RPT}}) & =B_{11.2}^{-1}+\mu_{11.2}\mu_{11.2}^{T}+2\mu_{11.2}^{T}\delta H_{p_{2}+2}\left(\chi_{p_{2}}^{2};\Delta \right)-\Phi H_{p_{2}+2}\left(\chi_{p_{2}}^{2};\Delta \right)\\ & +\delta \delta^{T}\left[2H_{p_{2}+2}\left(\chi_{p_{2}}^{2};\Delta \right)-H_{p_{2}+4}\left(\chi_{p_{2}}^{2};\Delta \right)\right],\\ Cov({\hat{\beta }}_{1}^{\mathrm{RSE}}) & =B_{11.2}^{-1}+\mu_{11.2}\mu_{11.2}^{T}+2\left(p_{2}-2\right)\mu_{11.2}^{T}\delta E\left(\chi_{p_{2}+2}^{-2}\left(\Delta \right)\right)\\ & -\left(p_{2}-2\right)\Phi \left\{2E\left(\chi_{p_{2}+2}^{-2}\left(\Delta \right)\right)-\left(p_{2}-2\right)E\left(\chi_{p_{2}+2}^{-4}\left(\Delta \right)\right)\right\}\\ & +\left(p_{2}-2\right)\delta \delta^{T}\left\{-2E\left(\chi_{p_{2}+4}^{-2}\left(\Delta \right)\right)+2E\left(\chi_{p_{2}+2}^{-2}\left(\Delta \right)\right)+\left(p_{2}-2\right)E\left(\chi_{p_{2}+4}^{-4}\left(\Delta \right)\right)\right\},\\ Cov({\hat{\beta }}_{1}^{\mathrm{RPS}}) & =\mathrm{Cov}\left({\hat{\beta }}_{1}^{\mathrm{RSE}}\right)+2\delta \mu_{11.2}^{T}E\left(\left\{1-\left(p_{2}-2\right)\chi_{p_{2}+2}^{-2}\left(\Delta \right)\right\}I\left(\chi_{p_{2}+2}^{2}\left(\Delta \right)\leq p_{2}-2\right)\right)\\ & -2\Phi E\left(\left\{1-\left(p_{2}-2\right)\chi_{p_{2}+2}^{-2}\left(\Delta \right)\right\}I\left(\chi_{p_{2}+2}^{2}\left(\Delta \right)\leq p_{2}-2\right)\right)\\ & -2\delta \delta^{T}E\left(\left\{1-\left(p_{2}-2\right)\chi_{p_{2}+4}^{-2}\left(\Delta \right)\right\}I\left(\chi_{p_{2}+4}^{2}\left(\Delta \right)\leq p_{2}-2\right)\right)\\ & +2\delta \delta^{T}E\left(\left\{1-\left(p_{2}-2\right)\chi_{p_{2}+2}^{-2}\left(\Delta \right)\right\}I\left(\chi_{p_{2}+2}^{2}\left(\Delta \right)\leq p_{2}-2\right)\right)\\ & -{\left(p_{2}-2\right)}^{2}\Phi E\left(\chi_{p_{2}+2}^{-4}\left(\Delta \right)I\left(\chi_{p_{2}+2,\alpha }^{2}\left(\Delta \right)\leq p_{2}-2\right)\right)\\ & -{\left(p_{2}-2\right)}^{2}\delta \delta^{T}E\left(\chi_{p_{2}+2,\alpha }^{-4}\left(\Delta \right)I\left(\chi_{p_{2}+2,\alpha }^{2}\left(\Delta \right)\leq p_{2}-2\right)\right)\\ & +\Phi H_{p_{2}+2}\left(p_{2}-2;\Delta \right)+\delta \delta^{T}H_{p_{2}+4}\left(p_{2}-2;\Delta \right). \end{array}$$

**Proof.**  See Appendix B.2. □

**Corollary** **2.** *Under the local alternatives* ($K_{n}$) *and from Theorem 3, the risk of the estimators are obtained as*

$$\begin{array}{cc} R\left[{\hat{\beta }}_{1}^{\mathrm{RFM}}\right] & =tr\left({QB}_{11.2}^{-1}\right)+\mu_{11.2}^{T}Q\mu_{11.2},\\ R[{\hat{\beta }}_{1}^{\mathrm{RSM}}] & =tr\left({QB}_{11}^{-1}\right)+\gamma^{T}Q\gamma ,\\ R\left[{\hat{\beta }}_{1}^{\mathrm{RPT}}\right] & =tr\left({QB}_{11.2}^{-1}\right)+\mu_{11.2}^{T}Q\mu_{11.2}+2\mu_{11.2}^{T}Q\delta H_{p_{2}+2}\left(\chi_{p_{2}}^{2};\Delta \right)\\ & -tr\left(Q\Phi \right)H_{p_{2}+2}\left(\chi_{p_{2}}^{2};\Delta \right)+\delta Q\delta^{T}\left[2H_{p_{2}+2}\left(\chi_{p_{2}}^{2};\Delta \right)-H_{p_{2}+4}\left(\chi_{p_{2}}^{2};\Delta \right)\right],\\ R\left[{\hat{\beta }}_{1}^{\mathrm{RSE}}\right] & =tr\left({QB}_{11.2}^{-1}\right)+\mu_{11.2}^{T}Q\mu_{11.2}+2\left(p_{2}-2\right)\mu_{11.2}^{T}Q\delta E\left(\chi_{p_{2}+2}^{-2}\left(\Delta \right)\right)\\ & -\left(p_{2}-2\right)\mathrm{tr}\left(Q\Phi \right)\left[E\left(\chi_{p_{2}+2}^{-2}\left(\Delta \right)\right)-\left(p_{2}-2\right)E\left(\chi_{p_{2}+2}^{-4}\left(\Delta \right)\right)\right]\\ & +\left(p_{2}-2\right)\delta^{T}Q\delta \left[2E\left(\chi_{p_{2}+2}^{-2}\left(\Delta \right)\right)-2E\left(\chi_{p_{2}+4}^{-2}\left(\Delta \right)\right)-\left(p_{2}-2\right)E\left(\chi_{p_{2}+4}^{-4}\left(\Delta \right)\right)\right],\\ R\left[{\hat{\beta }}_{1}^{\mathrm{RPS}}\right] & =R\left[{\hat{\beta }}_{1}^{\mathrm{RSE}}\right]+2\delta Q\mu_{11.2}^{T}E\left(\left\{1-\left(p_{2}-2\right)\chi_{p_{2}+2}^{-2}\left(\Delta \right)\right\}I\left(\chi_{p_{2}+2}^{2}\left(\Delta \right)\leq p_{2}-2\right)\right)\\ & -2tr\left(Q\Phi \right)E\left(\left\{1-\left(p_{2}-2\right)\chi_{p_{2}+2}^{-2}\left(\Delta \right)\right\}I\left(\chi_{p_{2}+2}^{2}\left(\Delta \right)\leq p_{2}-2\right)\right)\\ & -2\delta^{T}Q\delta E\left(\left\{1-\left(p_{2}-2\right)\chi_{p_{2}+4}^{-2}\left(\Delta \right)\right\}I\left(\chi_{p_{2}+4}^{2}\left(\Delta \right)\leq p_{2}-2\right)\right)\\ & +2\delta^{T}Q\delta E\left(\left\{1-\left(p_{2}-2\right)\chi_{p_{2}+2}^{-2}\left(\Delta \right)\right\}I\left(\chi_{p_{2}+2}^{2}\left(\Delta \right)\leq p_{2}-2\right)\right)\\ & -{\left(p_{2}-2\right)}^{2}tr\left(Q\Phi \right)E\left(\chi_{p_{2}+2}^{-4}\left(\Delta \right)I\left(\chi_{p_{2}+2}^{2}\left(\Delta \right)\leq p_{2}-2\right)\right)\\ & -{\left(p_{2}-2\right)}^{2}\delta^{T}Q\delta E\left(\chi_{p_{2}+2}^{-4}\left(\Delta \right)I\left(\chi_{p_{2}+2}^{2}\left(\Delta \right)\leq p_{2}-2\right)\right)\\ & +tr\left(Q\Phi \right)H_{p_{2}+2}\left(p_{2}-2;\Delta \right)+\delta^{T}Q\delta H_{p_{2}+4}\left(p_{2}-2;\Delta \right). \end{array}$$

From Theorem 2, when $B_{12}=0,$ the risks of estimators ${\hat{\beta }}_{1}^{\mathrm{RSM}},$${\hat{\beta }}_{1}^{\mathrm{RPT}},$${\hat{\beta }}_{1}^{\mathrm{RSE}},$ and ${\hat{\beta }}_{1}^{\mathrm{RPS}}$ are reduced to common value $\mathrm{tr}\left({QB}_{11.2}^{-1}\right)+\mu_{11.2}^{T}Q\mu_{11.2}$, the risk of ${\hat{\beta }}_{1}^{\mathrm{RFM}}$. If $B_{12}\neq 0$, the results can be summarized as follows:

1. The risk of ${\hat{\beta }}_{1}^{\mathrm{RFM}}$ remains constant while the risk of ${\hat{\beta }}_{1}^{\mathrm{RSM}}$ is an unbounded function of Δ since Δ∈[0,∞).
2. The risk of ${\hat{\beta }}_{1}^{\mathrm{RPT}}$ increases as Δ moves away from zero, achieves it maximum and then decreases towards the risk of the full model estimator.
3. The risk of ${\hat{\beta }}_{1}^{\mathrm{RFM}}$ is smaller than the risk of ${\hat{\beta }}_{1}^{\mathrm{RPT}}$ for small values in the neighborhood of Δ and for the rest of the parameter space, ${\hat{\beta }}_{1}^{\mathrm{RPT}}$ outperforms ${\hat{\beta }}_{1}^{\mathrm{RFM}}$, thus, $R\left[{\hat{\beta }}_{1}^{\mathrm{RFM}}\right]>R\left[{\hat{\beta }}_{1}^{\mathrm{RPT}}\right]$.
4. Comparing the risks of ${\hat{\beta }}_{1}^{\mathrm{RSE}}$ and ${\hat{\beta }}_{1}^{\mathrm{RFM}}$, it can be seen that the estimator ${\hat{\beta }}_{1}^{\mathrm{RSE}}$ outperforms ${\hat{\beta }}_{1}^{\mathrm{RFM}}$ that is, $R\left[{\hat{\beta }}_{1}^{\mathrm{RSE}}\right]\leq R\left[{\hat{\beta }}_{1}^{\mathrm{RFM}}\right]$ for all Δ≥0.

## 4. Simulation Studies

In this section, we conduct a simulation study to assess the performance of the suggested estimators for finite samples. The criterion for comparing the performance of any estimator in our study is the mean square error. We simulate the response from the following LMM model

(3) $$Y_{i}=X_{i}\beta +Z_{i}a_{i}+\epsilon_{i},$$

where $\epsilon_{i}∼N\left(0,\sigma^{2}I_{n_{i}}\right)$ with $\sigma^{2}=1$. We generate random effect covariate $a_{i}$ from a multivariate normal distribution with zero mean and covariance matrix $G=0.5I_{2\times 2}$, where $I_{2\times 2}$ is 2×2 identity matrix. The design matrix $X_{i}={\left(x_{i1},\ldots ,x_{in_{i}}\right)}^{\prime }$ is generated from a $n_{i}$-multivariate normal distribution with mean vector and covariance matrix $\sum_{x}$. Furthermore, we assume that the off-diagonal elements of the covariance matrix $\sum_{x}$ are equal to ρ, which is the coefficient of correlation between any two predictors, with ρ=0.3,0.7,0.9. The ratio of the largest eigenvalue to the smallest eigen-value of matrix $X^{T}V^{-1}X$ is calculated as a condition number index (CNI) [24], which assesses the existence of multicollinearity in the design matrix. If the CNI is larger than 30, then the model has significant multicollinearity. Our simulations are based on the linear mixed effects model in Equation (3) with n=60 and 100 subjects.

We consider a situation when the model is assumed to be sparse. In this study, our interest lies in testing the hypothesis $H_{o}:\beta_{2}=0$, and our goal is to estimate the fixed effect coefficient $\beta_{1}$. We partition the fixed effects coefficients as $\beta ={\left(\beta_{1}^{\prime },\beta_{2}^{\prime }\right)}^{\prime }={\left(\beta_{1}^{\prime },0_{p_{2}}\right)}^{\prime }.$ The coefficients $\beta_{1}$ and $\beta_{2}$ are $p_{1}$ and $p_{2}$ dimensional vectors, respectively, with $p=p_{1}+p_{2}$.

In order to investigate the behavior of the estimators, we define $\Delta^{*}=||\beta -\beta_{o}\left|\right|$, where $\beta_{o}={\left(\beta_{1}^{T},0_{p_{2}}\right)}^{T}$ and ||.|| is the euclidean norm. We considered $\Delta^{*}$ values between 0 and 4. If $\Delta^{*}=0$, then we will have $\beta ={\left(1,1,1,1,\underset{p_{2}}{\underset{︸}{0,0,\ldots ,0}}\right)}^{T}$ to generate the response under null hypothesis. On the other hand, when $\Delta^{*}\geq 0,$ say $\Delta^{*}=4,$ we will have $\beta ={\left(1,1,1,1,4,\underset{p_{2}-1}{\underset{︸}{0,0,\ldots ,0}}\right)}^{T}$ to generate the response under the local alternative hypothesis. In our simulation study, we consider the number of fixed effect or predictor variables as $\left(p_{1},p_{2}\right)\in \left\{\left(5,40\right),\left(5,500\right),\left(5,1000\right)\right\}.$ Each realization is repeated 5000 times to obtain consistent results and compute the MSE of suggested estimators with α=0.05.

Based on the simulated data, we calculate the mean square error (MSE) of all the estimators as MSE$\left(\hat{\beta }\right)=\frac{1}{5000}\sum_{j=1}^{5000}{\left(\hat{\beta }-\beta \right)}^{T}\left(\hat{\beta }-\beta \right)$, where $\hat{\beta }$ denotes any one of ${\hat{\beta }}^{\mathrm{RSM}},{\hat{\beta }}^{\mathrm{RPT}},{\hat{\beta }}^{\mathrm{RSE}}$ and ${\hat{\beta }}^{\mathrm{RPS}}$, in the jth repetition. We use the relative mean squared efficiency (RMSE), or the ratio of MSE for risk performance comparison. The RMSE of an estimator ${\hat{\beta }}^{*}$ with respect to the baseline full model ridge estimator ${\hat{\beta }}_{1}^{\mathrm{RFM}}$ is defined as $\mathrm{RMSE}\left({\hat{\beta }}_{1}^{\mathrm{RFM}}:{\hat{\beta }}_{1}^{*}\right)=\frac{\mathrm{MSE}({\hat{\beta }}_{1}^{\mathrm{RFM}})}{\mathrm{MSE}({\hat{\beta }}_{1}^{*})},$ where $\beta_{1}^{*}$ is one of the suggested estimators under consideration.

### 4.1. Simulation Results

**Figure 1:**
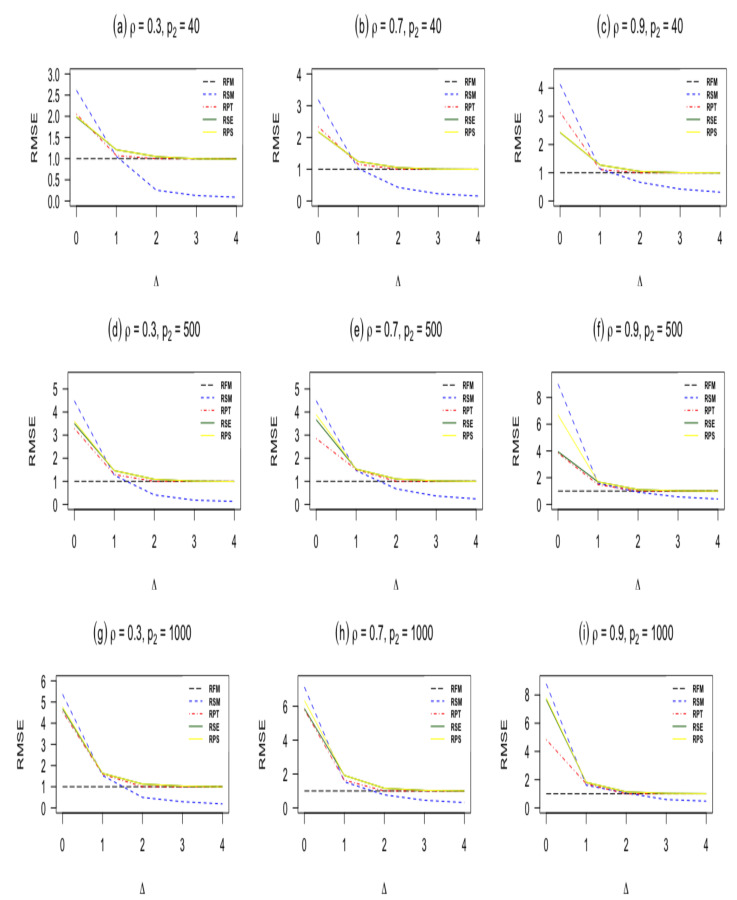
RMSE of estimators as a function of the non-centrality parameter Δ when *n* = 60, and $p_{1}=5$.

**Figure 2:**
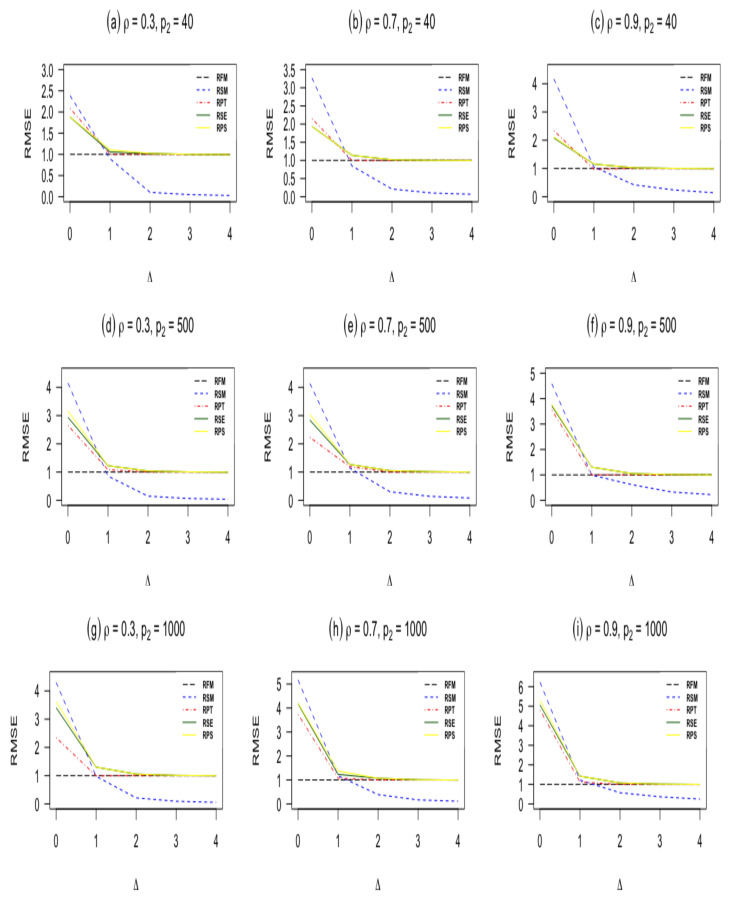
RMSE of estimators as a function of the non-centrality parameter Δ when *n* = 100, and $p_{1}=5$.

In this subsection, we present the results from our simulation study. We report the results for n=60,100 and $p_{1}=5$ with different values of correlation coefficient ρ are shown in Table 1. Furthermore, we plot the RMSEs against $\Delta^{*}$ in Figure 1 and Figure 2. The findings can be summarized as follows:

1. When $\Delta^{*}=0$, the sub-model RSM outperforms all other estimators. As $\Delta^{*}=0$ moves from zero, the RMSE of the sub-model decreases and goes to zero.
2. The pretest ridge estimator RPT outperforms shrinkage ridge and positive Stein ridge estimators in the case of $\Delta^{*}=0$. However, for large number of sparse predictors $p_{2}$ while keeping $p_{1}$ and *n* fixed, RPT is less efficient than RPS and RSE. In the case of $\Delta^{*}$ being larger than zero, the RMSE of RPT decreases, and it remains below 1 for immediate values of $\Delta^{*}$, after that the RMSE of RPT increases and approaches one for larger values of $\Delta^{*}.$
3. RPS performs better than RSE in the entire parameter space induced by $\Delta^{*}$ as presented in Table 1 and Table 2. Similarly, both shrinkage estimators RPS and RSE outperforms the full ridge model estimator irrespective of the corrected sub-model selected. This is consistent with the asymptotic theory presented in Section 3.
4. $\Delta^{*}$ which measures the degree of deviation from the Assumption 1 on the parameter space, it is clear that one cannot go wrong with the use of shrinkage estimators even if the selected sub-model is wrongly specified. As evident from Table 1 and Table 2, Figure 1 and Figure 2, if the selected sub-model is correct, that is, $\Delta^{*}=0$, then the shrinkage estimators are relatively efficient compared with the ridge full model estimator. On the other hand, if the sub-model is misspecified, the gain slowly diminishes. However, in terms of risk, the shrinkage estimators are at least as good as the full ridge model estimator. Therefore, the use of shrinkage estimators makes sense in application when a sub-model cannot be correctly specified.
5. The RMSE of the ridge-type estimators are an increasing function of the amount of multicollinearity. This indicates that the ridge-type estimators perform better than the classical estimator in the presence of multicollinearity among predictor variables.

### 4.2. Comparison with LASSO-Type Estimators

We compare our listed estimators with the LASSO and adaptive LASSO estimators. A 10-fold cross-validation is used for selecting the optimal value of the penalty parameters that minimizes the mean square errors for the LASSO-type estimators. The results for ρ=0.3,0.7,0.9, n=60,100, $p_{1}=10$ and $p_{2}=50,500,1000,2000$ are presented in Table 3. We observe the following from Table 3.

1. The performance of the sub-model estimator is the best among all estimators.
2. The pretest ridge estimator performs better than the other estimators. However, for larger values of sparse predictors $p_{2}$ the shrinkage estimators outperform the pretest estimator.
3. The performance of the LASSO and aLASSO estimators are comparable when ρ is small. The pretest and shrinkage estimators remain stable for a given value of ρ.
4. For a large number of sparse predictors $p_{2}$, the shrinkage and pretest ridge estimators outperforms the lasso-type estimators. This indicates the superiority of the shrinkage estimators over the LASSO-type estimators. Therefore shrinkage estimators are preferable when there is multicollinearity in our predictor variables.

## 5. Real Data Application

We consider two real data analyses using Amsterdam Growth and Health Data and a genetic and brain network connectivity edge weight data to illustrate the performance of the proposed estimators.

### 5.1. Amsterdam Growth and Health Data (AGHD)

The AGHD data is obtained from the Amsterdam Growth and Health Study [25]. The goal of this study is to investigate the relationship between lifestyle and health in adolescence into young adulthood. The response variable *Y* is the total serum cholesterol measured over six time points. There are five covariates: $X_{1}$ is the baseline fitness level measured as the maximum oxygen uptake on a treadmill, $X_{2}$ is the amount of body fat estimated by the sum of the thickness of four skinfolds, $X_{3}$ is a smoking indicator (0 = no, 1 = yes), $X_{4}$ is the gender (1 = female, 2 = male), and time measurement as $X_{5}$ and subject specific random effects.

A total of 147 subjects participated in the study where all variables were measured at $n_{i}=6$ time occasions. In order to apply the proposed methods, firstly, we apply a variable selection based on AIC procedure to select the sub-model. For the AGHD data, we fit a linear mixed model with all the five covariates for both fixed and subject specific random effects by two stage selection procedure for the purpose of choosing both the random and fixed effects. The analysis found $X_{2}$ and $X_{5}$ to be significant covariates for prediction of the response variable serum cholestrol and the other variables are ignored since they are not significantly important. Based on this information, a sub-model is chosen to be $X_{2}$ and $X_{5}$ and the full model includes all the covariates. We construct the shrinkage estimators from the full-model and sub-model. In terms of null hypothesis, the restriction can be written as $\beta_{2}=\left(\beta_{1},\beta_{3},\beta_{4}\right)=\left(0,0,0\right)$ with p=5, $p_{1}=2$ and $p_{2}=3$.

To evaluate the performance of the estimators, we obtain the mean square prediction error (MSPE) using bootstrap samples. We draw 1000 bootstrap samples of the 147 subjects from the data matrix $\left\{\left(Y_{ij},X_{ij}\right),i=1,2,\ldots ,147;j=1,2,\ldots ,6\right\}$. We then calculate the relative prediction error (RPE) of $\beta_{1}^{*}$ with respect to $\beta_{1}^{\mathrm{RFM}}$, the full model estimator. The RPE is defined as

$$\mathrm{RPE}\left({\hat{\beta }}_{1}^{\mathrm{RFM}}:{\hat{\beta }}_{1}^{*}\right)=\frac{\mathrm{MSPE}({\hat{\beta }}_{1}^{*})}{\mathrm{MSPE}({\hat{\beta }}_{1}^{\mathrm{RFM}})}=\frac{{\left(Y-X_{1}{\hat{\beta }}_{1}^{*}\right)}^{\prime }\left(Y-X_{1}{\hat{\beta }}_{1}^{*}\right)}{{\left(Y-X_{1}{\hat{\beta }}_{1}^{\mathrm{RFM}}\right)}^{\prime }\left(Y-X_{1}{\hat{\beta }}_{1}^{\mathrm{RFM}}\right)},$$

where $\beta_{1}^{*}$ is one of the listed estimators. If RPE<1, then ${\hat{\beta }}_{1}^{*}$ outperforms ${\hat{\beta }}_{1}^{\mathrm{RFM}}.$

Table 4 reports the estimates, standard error of the non-sparse predictors and RPEs of the estimators with respect to the full model. As expected, the sub-model ridge estimator ${\hat{\beta }}_{1}^{\mathrm{RSM}}$ has the minimum RPE because it is computed when the sub-model is correct, that is, $\Delta^{*}=0$. It is evident by the RPE values in Table 4 that the shrinkage estimators are superior to the LASSO-type estimators. Furthermore, the positive shrinkage is more efficient than the shrinkage ridge estimator.

### 5.2. Resting-State Effective Brain Connectivity and Genetic Data

This data comprises longitudinal resting-state functional magnetic resonance imaging (rs-fMRI) effective brain connectivity network and genetic study [26] data obtained from a sample of 111 subjects with a total of 319 rs-fMRI scans from the Alzheimer’s Disease Neuroimaging Initiative (ADNI) database. The 111 subjects comprise 36 cognitively normal (CN), 63 mild cognitive impairment (MCI) and 12 Alzheimer’s Disease (AD) subjects. The response is a network connection between regions of interest estimated from an rs-fMRI scan within the Default Mode Network (DMN), and we observe a longitudinal sequence of such connections for each subject with the number of repeated measurements. The DMN consists of a set of brain regions that tend to be active in resting-state, when a subject is mind wandering with no intended task. For this data analysis, we consider the network edge weight from the left intraparietal cortex to posterior cingulate cortex (LIPC → PCC) as our response. The genetic data are single nucleotide polymorphism (SNPs) from non-sex chromosomes, i.e., chromosome 1 to chromosome 22. SNPs with minor allele frequency less than 5% are removed as are SNPs with a Hardy–Weinberg equilibrium p-value lower than $10^{-6}$ or a missing rate greater than 5%. After preprocessing we are left with 1,220,955 SNPs and the longitudinal rs-fMRI effective connectivity network using the 111 subjects with rs-fMRI data. The response is network edge weight. There are SNPs which are the fixed effects and subject specific random effects.

In order to apply the proposed methods, we use a genome- wide association study (GWAS) for screening the genetic data to 100 SNPs. We implement a second screening by applying multinomial logistic regression to identify a smaller subset of the 100 SNPs that are potentially associated with disease (CN/MCI/AD). This yields a subset of top 10 SNPs. This showed the top 10 SNPs are the most important predictors and the other 90 SNPs are ignored as not significant. We now have two models, which are the full model with all 100 SNPs and sub-model with 10 SNPs selected. Finally, we construct the pretest and shrinkage estimators from the full-model and sub-model.

We draw 1000 bootstrap samples with replacements from the corresponding data matrix $\{\left(Y_{ij},X_{ij}\right),i=1,\ldots ,111;j=1,\ldots ,n_{i}\}.$ We report the RPE of the estimators based on the bootstrap simulation with respect to the full model ridge estimator in Table 5. We observe that the RPE of the sub-model, pretest, shrinkage and positive shrinkage ridge estimators outperforms the full model estimator. Clearly, the sub-model ridge estimator has the smallest RPE since it’s computed when the candidate sub-model is correct, i.e., Δ=0. Both shrinkage ridge estimators outperform the pretest ridge estimator. Particularly, the positive shrinkage performed better than the shrinkage estimator. The performance of both shrinkage and pretest ridge estimators are better than the LASSO-type estimators. Thus, the data analysis is in line with our simulation and theoretical findings.

## 6. Conclusions

In this paper, we present efficient estimation strategies for the linear mixed effect model when there exists multicollinearity among predictor variables for high-dimensional data application. We considered the estimation of fixed effects parameters in the linear mixed model when some of the predictors may have a very weak influence on the response of interest. We introduced pretest and shrinkage estimation in our model using the ridge estimation as the reference estimator. In addition, we established the asymptotic properties of the pretest and shrinkage ridge estimators. Our theoretical findings demonstrate that the shrinkage ridge estimators outperform the full model ridge estimator and perform relatively better than the sub-model estimator in a wide range of the parameter space.

Additionally, a Monte Carlo simulation was conducted to investigate and assess the finite sample behavior of proposed estimators when the model is sparse (restrictions on parameters hold). As expected, the sub-model ridge estimator outshines all other estimators when the restrictions hold. However, when this assumption is violated, the shrinkage and pretest ridge estimators outperform the sub-model estimator. Furthermore, when the number of sparse predictors are extremely large relative to the sample size, the shrinkage estimators outperform the pretest ridge estimator. These numerical results are consistent with our asymptotic result. We also assess the relative performance of the LASSO-type estimators with our ridge-type estimators. We observe that the performance of pretest and shrinkage ridge estimators are superior to the LASSO-type estimators when predictors are highly correlated. For our real data application, the shrinkage ridge estimators are superior with the smallest relative prediction error compared to the LASSO-type estimators.

In summary, the results of the data analyses strongly confirm the findings of the simulation study and suggest the use of the shrinkage ridge estimation strategy when no prior information about the parameter subspace is available. The results of our simulation study and real data application are consistent with available results in [27,28,29].

In our future work, we will focus on other penalty estimators like the Elastic-Net, the minimax concave penalty (MCP), and the smoothly clipped absolute deviation method (SCAD) as estimation strategy in LMM for high-dimensional data. These estimators will be assessed and compared with the proposed ridge-type estimators. Another interesting extension will be integrating two sub-models by incorporating ridge-type estimation strategies in the linear mixed effect models. The goal is to improve the estimation accuracy of the non-sparse set of the fixed effects parameters by combining an over-fitted model estimator with an under-fitted one [27,29]. This approach will include combining two sub-models produced by two different variable selection techniques from the LMM [28].

## Figures and Tables

**Table 1:** RMSEs of RSM, RPT, RSE, and RPS estimators with respect to ${\hat{\beta }}^{\mathrm{RFM}}$ when Δ≥0 for $p_{1}=5$ and n=60.

ρ	$p_{2}$	Δ	CNI	RSM	RPT	RSE	RPS
0.3	40	0	361	2.61	2.07	1.94	1.96
		1		1.05	1.07	1.20	1.25
		2		0.25	0.95	1.04	1.05
		3		0.12	0.98	0.99	1.00
		4		0.08	1.00	1.00	1.00
	500	0	613	4.48	3.29	3.48	1.96
		1		1.26	1.12	1.26	1.29
		2		0.41	0.97	1.08	1.09
		3		0.18	0.99	1.00	1.00
		4		0.13	1.00	1.00	1.00
	1000	0	693	5.36	4.53	4.67	4.71
		1		1.53	1.21	1.35	1.39
		2		0.49	1.01	1.13	1.14
		3		0.28	0.99	0.99	0.99
		4		0.10	1.00	1.00	1.00
0.7	40	0	1352	3.18	2.33	2.17	2.18
		1		1.04	1.11	1.20	1.23
		2		0.42	1.03	1.04	1.04
		3		0.23	0.98	0.99	1.00
		4		0.14	1.00	1.00	1.00
	500	0	1789	4.48	2.76	2.94	3.02
		1		1.08	1.43	1.52	1.53
		2		0.67	1.03	1.07	1.06
		3		0.35	0.98	1.00	1.00
		4		0.19	1.00	1.00	1.00
	1000	0	2134	6.82	5.24	5.30	3.02
		1		1.16	1.32	1.42	1.53
		2		0.75	1.10	1.15	1.16
		3		0.39	0.99	1.00	1.00
		4		0.11	1.00	1.00	1.00

**Table 2:** RMSEs of RSM, RPT, RSE, and RPS estimators with respect to ${\hat{\beta }}^{\mathrm{RFM}}$ when Δ≥0 for $p_{1}=5$, and n=100.

ρ	$p_{2}$	Δ	CNI	RSM	RPT	RSE	RPS
0.3	40	0	150	2.38	2.09	1.88	1.90
		1		0.89	1.01	1.05	1.08
		2		0.21	0.94	1.01	1.02
		3		0.06	0.94	0.99	1.00
		4		0.02	1.00	1.00	1.00
	500	0	340	4.15	2.65	2.99	3.17
		1		0.87	1.08	1.18	1.21
		2		0.14	0.96	1.03	1.05
		3		0.06	0.99	0.99	1.00
		4		0.03	1.00	1.00	1.00
	1000	0	536	4.30	2.75	3.02	3.08
		1		0.96	1.09	1.13	1.15
		2		0.21	0.8	1.03	1.03
		3		0.09	1.00	1.00	1.00
		4		0.04	1.00	1.00	1.00
0.7	40	0	997	3.27	2.15	2.09	2.11
		1		0.85	1.02	1.09	1.10
		2		0.21	0.98	1.02	1.02
		3		0.06	0.99	0.99	0.99
		4		0.01	1.00	1.00	1.00
	500	0	1589	4.13	2.22	2.35	2.39
		1		1.04	1.19	1.21	1.20
		2		0.30	0.97	1.05	1.05
		3		0.14	1.00	1.00	1.00
		4		0.08	1.00	1.00	1.00
	1000	0	1751	5.17	3.71	4.03	4.09
		1		1.01	1.15	1.24	1.25
		2		0.39	1.04	1.07	1.06
		3		0.16	0.99	1.00	1.00
		4		0.11	1.00	1.00	1.00

**Table 3:** RMSEs of estimators with respect to ${\hat{\beta }}^{\mathrm{RFM}}$ when Δ=0 for $p_{1}=10$.

n	ρ	$p_{2}$	CNI	RSM	RPT	RSE	RPS	LASSO	aLASSO
60	0.3	50	35.64	3.31	2.25	1.82	1.95	1.23	1.28
		500	452.76	4.13	3.71	2.61	3.01	1.47	1.52
		1000	1265.34	5.02	4.28	4.61	4.78	1.96	2.15
		2000	4567.56	7.13	5.10	6.18	6.39	2.70	3.06
	0.7	50	61.34	3.52	3.05	2.51	2.55	1.14	1.21
		500	743.17	4.49	3.65	3.41	3.50	1.36	1.58
		1000	2350.89	5.84	4.11	4.32	4.61	1.68	1.95
		2000	6908.39	8.10	5.31	6.24	6.29	1.84	2.02
	0.9	50	120.21	4.21	3.61	3.34	3.35	1.10	1.05
		500	950.98	4.82	3.3.8	3.72	3.73	1.21	1.16
		1000	5892.51	6.35	4.10	5.01	5.13	1.42	1.31
		2000	8352.73	8.51	4.63	5.24	5.38	1.61	1.35
100	0.3	50	31.21	2.91	2.54	2.12	2.23	1.32	1.36
		500	356.64	3.75	3.31	2.84	2.92	1.54	1.61
		1000	975.32	4.25	2.53	3.42	3.61	1.92	2.06
		2000	2764.84	5.61	4.25	4.91	5.08	2.31	2.46
	0.7	50	52.79	3.18	2.61	2.30	2.37	1.28	1.53
		500	578.43	4.28	3.05	3.52	3.59	1.46	2.07
		1000	1281.66	5.10	3.26	3.78	3.82	1.84	2.52
		2000	3498.30	6.12	3.01	4.26	4.33	2.27	2.41
	0.9	50	79.41	4.11	3.41	3.21	3.28	1.28	1.21
		500	681.43	4.35	3.55	3.41	3.50	1.43	1.51
		1000	1470.32	5.82	3.18	4.01	4.14	1.72	1.79
		2000	4105.90	7.04	4.57	5.22	5.32	1.87	1.96

**Table 4:** Estimate, standard error for the active predictors and RPEs of estimators with respect to full-model estimator for the Amsterdam Growth and Health Study data.

	RFM	RSM	RPT	RSE	RPS	LASSO	aLASSO
Estimate($\beta_{2}$ )	0.381	0.395	0.392	0.389	0.390	0.624	0.611
Standard error	0.104	0.102	0.100	0.009	0.008	0.081	0.079
Estimate ($\beta_{5}$)	0.137	0.125	0.131	0.130	0.133	0.101	0.105
Standard error	0.012	0.010	0.009	0.011	0.010	0.013	0.012
RPE	1.000	0.723	0.841	0.838	0.831	0.986	0.973

**Table 5:** RPEs of estimators.

	RFM	RSM	RPT	RSE	RPS	LASSO	aLASSO
RPE	1.000	0.802	0.947	0.932	0.928	1.051	1.190

## Data Availability

Publicly available datasets were analyzed in this study. This data can be found here https://pubmed.ncbi.nlm.nih.gov/22434862/ (accessed on 20 April 2021).

## Appendix A

**Proof** **of** **Proposition** **1.** The asymptotic relationship between the sub-model and full model estimators of $\beta_{1}$, we use the argument and equation: $\hat{Y}=Y-X_{2}{\hat{\beta }}_{2}^{\mathrm{RFM}}$, where

$$\begin{array}{cc} {\hat{\beta }}_{1}^{\mathrm{RFM}} & =\arg\min_{\beta_{1}}\left\{{\left(\hat{Y}-X_{1}\beta_{1}\right)}^{T}V^{-1}\left(\hat{Y}-X_{1}\beta_{1}\right)+\lambda ||\beta_{1}{\left|\right|}^{2}\right\}\\ \; & ={\left[X_{1}^{T}V^{-1}X_{1}+\lambda I_{p_{1}}\right]}^{-1}X_{1}^{T}V^{-1}\hat{Y}\\ \; & ={\left[X_{1}^{T}V^{-1}X_{1}+\lambda I_{p_{1}}\right]}^{-1}X_{1}^{T}V^{-1}Y-{\left[X_{1}^{T}V^{-1}X_{1}+\lambda I_{p_{1}}\right]}^{-1}X_{1}^{T}V^{-1}X_{2}{\hat{\beta }}_{2}^{\mathrm{RFM}}\\ \; & ={\hat{\beta }}_{1}^{\mathrm{RSM}}-{\left[X_{1}V^{-1}X_{1}+\lambda I_{p_{1}}\right]}^{-1}X_{1}^{T}V^{-1}X_{2}{\hat{\beta }}_{2}^{\mathrm{RFM}}\\ \; & ={\hat{\beta }}_{1}^{\mathrm{RSM}}-B_{11}^{-1}B_{12}{\hat{\beta }}_{2}^{\mathrm{RFM}} \end{array}$$

From Theorem 1, we partition $\sqrt{n}\left({\hat{\beta }}^{\mathrm{RFM}}-\beta \right)$ as $\sqrt{n}\left({\hat{\beta }}^{\mathrm{RFM}}-\beta \right)=\left(\sqrt{n}\left({\hat{\beta }}_{1}^{\mathrm{RFM}}-\beta_{1}\right),\sqrt{n}\left({\hat{\beta }}_{2}^{\mathrm{RFM}}-\beta_{2}\right)\right)$. We obtain $\sqrt{n}\left({\hat{\beta }}_{1}^{\mathrm{RFM}}-\beta_{1}\right)\overset{D}{\to }N_{p_{1}}\left(-\mu_{11.2},B_{11.2}^{-1}\right),$ where $B_{11.2}^{-1}=B_{11}-B_{12}B_{22}^{-1}B_{21}.$ We have shown that ${\hat{\beta }}_{1}^{\mathrm{RSM}}={\hat{\beta }}_{1}^{\mathrm{RFM}}+B_{11}^{-1}B_{12}{\hat{\beta }}_{2}^{\mathrm{RFM}}.$ Using this expression and under the local alternative $\left\{K_{n}\right\}$, we obtain the following expressions

$$\begin{array}{cc} \varphi_{2} & =\sqrt{n}\left({\hat{\beta }}_{1}^{\mathrm{RSM}}-\beta_{1}\right)\\ \; & =\sqrt{n}\left({\hat{\beta }}_{1}^{\mathrm{RFM}}+B_{11}^{-1}B_{12}{\hat{\beta }}_{2}^{\mathrm{RFM}}-\beta_{1}\right)\\ \; & =\varphi_{1}+B_{11}^{-1}B_{12}\sqrt{n}{\hat{\beta }}_{2}^{\mathrm{RFM}},\\ \varphi_{3} & =\sqrt{n}\left({\hat{\beta }}_{1}^{\mathrm{RFM}}-{\hat{\beta }}_{1}^{\mathrm{RSM}}\right)\\ \; & =\sqrt{n}\left({\hat{\beta }}_{1}^{\mathrm{RFM}}-\beta_{1}\right)-\sqrt{n}\left({\hat{\beta }}_{1}^{\mathrm{RSM}}-\beta_{1}\right)\\ \; & =\varphi_{1}-\varphi_{2}. \end{array}$$

Since $\varphi_{2}$ and $\varphi_{3}$ are linear functions of $\varphi_{1},$ as n→∞, they are also asymptotically normally distributed. Their mean vectors and covariance matrices are as follows:

$$\begin{array}{cc} E(\varphi_{1}) & =E\left(\sqrt{n}\left({\hat{\beta }}_{1}^{\mathrm{RFM}}-\beta_{1}\right)\right)=-\mu_{11.2}\\ E(\varphi_{2}) & =E\left(\varphi_{1}+B_{11}^{-1}B_{12}\sqrt{n}{\hat{\beta }}_{2}^{\mathrm{RFM}}\right)\\ \; & =E\left(\varphi_{1}\right)+B_{11}^{-1}B_{12}\sqrt{n}E\left({\hat{\beta }}_{2}^{\mathrm{RFM}}\right)\\ \; & =-\mu_{11.2}+B_{11}^{-1}B_{12}\kappa =-\left(\mu_{11.2}-\delta \right)=-\gamma \\ E(\varphi_{3}) & =E\left(\varphi_{1}-\varphi_{2}\right)=-\mu_{11.2}-\left(-\left(\mu_{11.2}-\delta \right)\right)=\delta \\ Var(\varphi_{1}) & =B_{22.1}^{-1}\\ Var(\varphi_{2}) & =Var\left(\varphi_{1}+B_{11}^{-1}B_{12}\sqrt{n}{\hat{\beta }}_{2}^{\mathrm{RFM}}\right)\\ \; & =Var\left(\varphi_{1}\right)+B_{11}^{-1}B_{12}B_{22.1}^{-1}B_{21}B_{11}^{-1}\\ \; & +2Cov\left[\sqrt{n}\left({\hat{\beta }}_{1}^{\mathrm{RFM}}-\beta_{1}\right),\sqrt{n}\left({\hat{\beta }}_{2}^{\mathrm{RFM}}-\beta_{2}\right)\right]{\left(B_{11}^{-1}B_{12}\right)}^{T}\\ \; & =B_{22.1}^{-1}-B_{11}^{-1}B_{12}B_{22.1}^{-1}B_{21}B_{11}^{-1}=B_{11}^{-1}\\ Var(\varphi_{3}) & =Var\left(\sqrt{n}\left({\hat{\beta }}_{1}^{\mathrm{RFM}}-{\hat{\beta }}_{1}^{\mathrm{RSM}}\right)\right)\\ \; & =Var\left(\sqrt{n}\left({\hat{\beta }}_{1}^{\mathrm{RFM}}-{\hat{\beta }}_{1}^{\mathrm{RFM}}-B_{11}^{-1}B_{12}{\hat{\beta }}_{2}^{\mathrm{RFM}}\right)\right)\\ \; & =B_{11}^{-1}B_{12}Var\left[\sqrt{n}{\hat{\beta }}_{2}^{\mathrm{RFM}}\right]{\left(B_{11}^{-1}B_{12}\right)}^{T}\\ \; & =B_{11}^{-1}B_{12}B_{22.1}^{-1}B_{21}B_{11}^{-1}=\Phi \\ Cov(\varphi_{1},\varphi_{3}) & =Cov\left[\sqrt{n}\left({\hat{\beta }}_{1}^{\mathrm{RFM}}-\beta_{1}\right),\sqrt{n}\left({\hat{\beta }}_{1}^{\mathrm{RFM}}-{\hat{\beta }}_{1}^{\mathrm{RSM}}\right)\right]\\ \; & =Var\left(\sqrt{n}\left({\hat{\beta }}_{1}^{\mathrm{RFM}}-\beta_{1}\right)\right)-Cov\left[\sqrt{n}\left({\hat{\beta }}_{1}^{\mathrm{RFM}}-\beta_{1}\right),\sqrt{n}\left({\hat{\beta }}_{1}^{\mathrm{RSM}}-\beta_{1}\right)\right]\\ \; & =Var\left(\varphi_{1}\right)-Cov\left[\sqrt{n}\left({\hat{\beta }}_{1}^{\mathrm{RFM}}-\beta_{1}\right),\sqrt{n}\left({\hat{\beta }}_{1}^{\mathrm{RFM}}-\beta_{1}\right)+\sqrt{n}B_{11}^{-1}B_{12}{\hat{\beta }}_{2}^{\mathrm{RFM}}\right]\\ \; & =B_{11}^{-1}B_{12}B_{22.1}^{-1}B_{21}B_{11}^{-1}=\Phi \end{array}$$

$$\begin{array}{cc} Cov(\varphi_{2},\varphi_{3}) & =Cov\left[\sqrt{n}\left({\hat{\beta }}_{1}^{\mathrm{RSM}}-\beta_{1}\right),\sqrt{n}\left({\hat{\beta }}_{1}^{\mathrm{RFM}}-{\hat{\beta }}_{1}^{\mathrm{RSM}}\right)\right]\\ \; & =Cov\left[\sqrt{n}\left({\hat{\beta }}_{1}^{\mathrm{RSM}}-\beta_{1}\right),\sqrt{n}\left({\hat{\beta }}_{1}^{\mathrm{RFM}}-\beta_{1}\right)\right]-Var\left(\sqrt{n}\left({\hat{\beta }}_{1}^{\mathrm{RSM}}-\beta_{1}\right)\right)\\ \; & =B_{11.2}^{-1}-B_{11}^{-1}B_{12}B_{22.1}^{-1}B_{21}B_{11}^{-1}-B_{11}^{-1}\\ \; & =B_{11.2}^{-1}-\left(B_{11.2}^{-1}-B_{11}^{-1}\right)-B_{11}^{-1}=0 \end{array}$$

Therefore, the asymptotic distributions of the vectors $\varphi_{2}$ and $\varphi_{3}$ are obtained as follows:

$$\begin{array}{c} \varphi_{2}=\sqrt{n}\left({\hat{\beta }}_{1}^{\mathrm{RSM}}-\beta_{1}\right)\overset{D}{\to }N_{p_{1}}\left(-\gamma ,B_{11}^{-1}\right)\\ \varphi_{3}=\sqrt{n}\left({\hat{\beta }}_{1}^{\mathrm{RFM}}-{\hat{\beta }}_{1}^{\mathrm{RSM}}\right)\overset{D}{\to }N_{p_{1}}\left(\delta ,\Phi \right) \end{array}$$

  □

## Appendix B

We next introduce the lemmas given in [30] to aid with the proof of the bias and covariance of the estimators.

**Lemma** **A1.** *Let*$V={\left(V_{1},V_{2},\ldots V_{p}\right)}^{T}$ be a p-dimensional normal vector distributed as $N_{p}\left(\mu_{v},\sum_{p}\right),$ *then for a measurable function* Ψ, *we have*

$$\begin{array}{c} E\left[V\Psi (V^{T}V)\right]=\mu_{v}E\left[\Psi \chi_{p+2}^{2}\left(\Delta \right)\right]\\ E\left[{\mathrm{VV}}^{T}\Psi \left(V^{T}V\right)\right]=\sum_{p}E\left[\Psi \chi_{p+2}^{2}\left(\Delta \right)\right]+\mu_{v}\mu_{v}^{T}E\left[\Psi \chi_{p+4}^{2}\left(\Delta \right)\right] \end{array}$$

*where* $\chi_{k}^{2}\left(\Delta \right)$ *is a non-central chi-square distribution with k degrees of freedom and non-centrality parameter* Δ.

### Appendix B.1

**Proof** **of** **Theorem 2.** 

$$\begin{array}{cc} \mathrm{ADB}({\hat{\beta }}_{1}^{\mathrm{RFM}}) & =E\left\{\lim_{n\to \infty }\sqrt{n}\left({\hat{\beta }}_{1}^{\mathrm{RFM}}-\beta_{1}\right)\right\}\\ \; & =-\mu_{11.2}. \end{array}$$

$$\begin{array}{cc} \mathrm{ADB}({\hat{\beta }}_{1}^{\mathrm{RSM}}) & =E\left\{\lim_{n\to \infty }\sqrt{n}\left({\hat{\beta }}_{1}^{\mathrm{RSM}}-\beta_{1}\right)\right\}\\ \; & =E\left\{\lim_{n\to \infty }\sqrt{n}\left({\hat{\beta }}_{1}^{\mathrm{RFM}}-B_{11}^{-1}B_{12}{\hat{\beta }}_{2}^{\mathrm{RFM}}-\beta_{1}\right)\right\}\\ \; & =E\left\{\lim_{n\to \infty }\sqrt{n}\left({\hat{\beta }}_{1}^{\mathrm{RFM}}-\beta_{1}\right)\right\}-E\left\{\lim_{n\to \infty }\sqrt{n}\left(B_{11}^{-1}B_{12}{\hat{\beta }}_{2}^{\mathrm{RFM}}\right)\right\}\\ \; & =-\mu_{11.2}-E\left\{\lim_{n\to \infty }\sqrt{n}\left(B_{11}^{-1}B_{12}{\hat{\beta }}_{2}^{\mathrm{RFM}}\right)\right\}\\ \; & =-\mu_{11.2}-B_{11}^{-1}B_{12}\kappa =-\left(\mu_{11.2}+\delta \right)=-\gamma . \end{array}$$

Using Lemma 1,

$$\begin{array}{cc} \mathrm{ADB}({\hat{\beta }}_{1}^{\mathrm{RPT}}) & =E\left\{\lim_{n\to \infty }\sqrt{n}\left({\hat{\beta }}_{1}^{\mathrm{RPT}}-\beta_{1}\right)\right\}\\ \; & =E\left\{\lim_{n\to \infty }\sqrt{n}\left({\hat{\beta }}_{1}^{\mathrm{RFM}}-\left({\hat{\beta }}_{1}^{\mathrm{RFM}}-{\hat{\beta }}_{1}^{\mathrm{RSM}}\right)I\left(L_{n}\leq d_{n,\alpha }\right)-\beta_{1}\right)\right\}\\ \; & =E\left\{\lim_{n\to \infty }\sqrt{n}\left({\hat{\beta }}_{1}^{\mathrm{RFM}}-\beta_{1}\right)\right\}-E\left\{\lim_{n\to \infty }\sqrt{n}\left({\hat{\beta }}_{1}^{\mathrm{RFM}}-{\hat{\beta }}_{1}^{\mathrm{RSM}}\right)I\left(L_{n}\leq d_{n,\alpha }\right)\right\}\\ \; & =-\mu_{11.2}-E\left\{\lim_{n\to \infty }\sqrt{n}\left({\hat{\beta }}_{1}^{\mathrm{RFM}}-{\hat{\beta }}_{1}^{\mathrm{RSM}}\right)I\left(L_{n}\leq d_{n,\alpha }\right))\right\}\\ \; & =-\mu_{11.2}-\delta H_{p_{2}+2}\left(\chi_{p_{2}}^{2};\Delta \right). \end{array}$$

$$\begin{array}{cc} \mathrm{ADB}({\hat{\beta }}_{1}^{\mathrm{RSE}}) & =E\left\{\lim_{n\to \infty }\sqrt{n}\left({\hat{\beta }}_{1}^{\mathrm{RSE}}-\beta_{1}\right)\right\}\\ \; & =E\left\{\lim_{n\to \infty }\sqrt{n}\left({\hat{\beta }}_{1}^{\mathrm{RFM}}-\left({\hat{\beta }}_{1}^{\mathrm{RFM}}-{\hat{\beta }}_{1}^{\mathrm{RSM}}\right)\left(p_{2}-2\right)L_{n}^{-1}-\beta_{1}\right)\right\}\\ \; & =E\left\{\lim_{n\to \infty }\sqrt{n}\left({\hat{\beta }}_{1}^{\mathrm{RFM}}-\beta_{1}\right)\right\}-E\left\{\lim_{n\to \infty }\sqrt{n}\left({\hat{\beta }}_{1}^{\mathrm{RFM}}-{\hat{\beta }}_{1}^{\mathrm{RSM}}\right)\left(p_{2}-2\right)L_{n}^{-1}\right\}\\ \; & =-\mu_{11.2}-E\left\{\lim_{n\to \infty }\sqrt{n}\left({\hat{\beta }}_{1}^{\mathrm{RFM}}-{\hat{\beta }}_{1}^{\mathrm{RSM}}\right)\left(p_{2}-2\right)L_{n}^{-1}\right\}\\ \; & =-\mu_{11.2}-\left(p_{2}-2\right)\delta E\left(\chi_{p_{2}+2}^{-2}\left(\Delta \right)\right). \end{array}$$

$$\begin{array}{cc} \mathrm{ADB}({\hat{\beta }}_{1}^{\mathrm{RPS}}) & =E\left\{\lim_{n\to \infty }\sqrt{n}\left({\hat{\beta }}_{1}^{\mathrm{RPS}}-\beta_{1}\right)\right\}\\ \; & =E\left\{\lim_{n\to \infty }\sqrt{n}\left({\hat{\beta }}_{1}^{\mathrm{RSM}}+\left({\hat{\beta }}_{1}^{\mathrm{RFM}}-{\hat{\beta }}_{1}^{\mathrm{RSM}}\right)\left(1-\left(p_{2}-2\right)L_{n}^{-1}\right)I\left(L_{n}>p_{2}-2\right)-\beta_{1}\right)\right\}\\ \; & =E\{\sqrt{n}[{\hat{\beta }}_{1}^{\mathrm{RSM}}+\left({\hat{\beta }}_{1}^{\mathrm{RFM}}-{\hat{\beta }}_{1}^{\mathrm{RSM}}\right)\left(1-I\left(L_{n}\leq p_{2}-2\right)\right)\\ & -\left({\hat{\beta }}_{1}^{\mathrm{RFM}}-{\hat{\beta }}_{1}^{\mathrm{RSM}}\right)\left(p_{2}-2\right)L_{n}^{-1}I\left(L_{n}>p_{2}-2\right)-\beta_{1}]\}\\ \; & =E\left\{\lim_{n\to \infty }\sqrt{n}\left({\hat{\beta }}_{1}^{\mathrm{RFM}}-\beta_{1}\right)\right\}-E\left\{\lim_{n\to \infty }\sqrt{n}\left({\hat{\beta }}_{1}^{\mathrm{RFM}}-{\hat{\beta }}_{1}^{\mathrm{RSM}}\right)\left(p_{2}-2\right)I\left(L_{n}\leq p_{2}-2\right)\right\}\\ \; & -E\{\lim_{n\to \infty }\sqrt{n}\left({\hat{\beta }}_{1}^{\mathrm{RFM}}-{\hat{\beta }}_{1}^{\mathrm{RSM}}\right)\left(p_{2}-2\right)L_{n}^{-1}I\left(L_{n}>p_{2}-2\right)\\ \; & =-\mu_{11.2}-\delta H_{p_{2}+2}\left(\chi_{p_{2}-2}^{2};\Delta \right)\}-\left(p_{2}-2\right)\delta E\left\{\chi_{p_{2}+2}^{-2}\left(\Delta \right)I\left(\chi_{p_{2}+2}^{-2}>p_{2}-2\right)\right\}. \end{array}$$

    □

### Appendix B.2

In order to compute the risk functions, we first compute the asymptotic covariance of the estimators. The asymptotic covariance of an estimator ${\hat{\beta }}_{1}^{*}$ is expressed as

$$\mathrm{Cov}\left({\hat{\beta }}_{1}^{*}\right)=\lim_{n\to \infty }E\left\{n\left({\hat{\beta }}_{1}^{*}-\beta_{1}\right){\left({\hat{\beta }}_{1}^{*}-\beta_{1}\right)}^{T}\right\}.$$

**Proof** **of** **Theorem** **3.** We first start by computing the asymptotic covariance of the estimator ${\hat{\beta }}_{1}^{\mathrm{RFM}}$ as:

$$\begin{array}{cc} \mathrm{Cov}({\hat{\beta }}_{1}^{\mathrm{RFM}}) & =E\{\lim_{n\to \infty }\sqrt{n}\left({\hat{\beta }}_{1}^{\mathrm{RFM}}-\beta_{1}\right)\sqrt{n}{\left({\hat{\beta }}_{1}^{\mathrm{RFM}}-\beta_{1}\right)}^{T}\}\\ \; & =E\left(\varphi_{1}\varphi_{1}^{T}\right)=\mathrm{Cov}\left(\varphi_{1}\varphi_{1}^{T}\right)+E\left(\varphi_{1}\right)E\left(\varphi_{1}^{T}\right)\\ \; & =B_{11.2}^{-1}+\mu_{11.2}\mu_{11.2}^{T}. \end{array}$$

Furthermore, similarly, the asymptotic covariance of the estimator ${\hat{\beta }}_{1}^{\mathrm{RSM}}$ is obtained as:

$$\begin{array}{cc} \mathrm{Cov}({\hat{\beta }}_{1}^{\mathrm{RSM}}) & =E\{\lim_{n\to \infty }\sqrt{n}\left({\hat{\beta }}_{1}^{\mathrm{RSM}}-\beta_{1}\right)\sqrt{n}{\left({\hat{\beta }}_{1}^{\mathrm{RSM}}-\beta_{1}\right)}^{T}\}\\ \; & =E\left(\varphi_{2}\varphi_{2}^{T}\right)=Cov\left(\varphi_{2}\varphi_{2}^{T}\right)+E\left(\varphi_{2}\right)E\left(\varphi_{2}^{T}\right)\\ \; & =B_{11}^{-1}+\gamma \gamma^{T}. \end{array}$$

The asymptotic covariance of the estimator ${\hat{\beta }}_{1}^{\mathrm{RPT}}$ is obtained as:

$$\begin{array}{cc} \mathrm{Cov}({\hat{\beta }}_{1}^{\mathrm{RPT}}) & =E\{\lim_{n\to \infty }\sqrt{n}\left({\hat{\beta }}_{1}^{\mathrm{RPT}}-\beta_{1}\right)\sqrt{n}{\left({\hat{\beta }}_{1}^{\mathrm{RPT}}-\beta_{1}\right)}^{T}\}\\ \; & =E\{\lim_{n\to \infty }n[\left({\hat{\beta }}_{1}^{\mathrm{RFM}}-\beta_{1})-\left({\hat{\beta }}_{1}^{\mathrm{RFM}}-{\hat{\beta }}_{1}^{\mathrm{RSM}}\right)I\left(L_{n}\leq d_{n,\alpha }\right)\right]\\ \; & \left[{\left({\hat{\beta }}_{1}^{\mathrm{RFM}}-\beta_{1})-\left({\hat{\beta }}_{1}^{\mathrm{RFM}}-{\hat{\beta }}_{1}^{\mathrm{RSM}}\right)I\left(L_{n}\leq d_{n,\alpha }\right)\right]}^{T}\right\}\\ \; & =E\left\{\left[\varphi_{1}-\varphi_{3}I\left(L_{n}\leq d_{n,\alpha }\right)\right]{\left[\varphi_{1}-\varphi_{3}I\left(L_{n}\leq d_{n,\alpha }\right)\right]}^{T}\right\}\\ \; & =E\left\{\varphi_{1}\varphi_{1}^{T}-2\varphi_{3}\varphi_{1}^{T}I\left(L_{n}\leq d_{n,\alpha }\right)+\varphi_{3}\varphi_{3}^{T}I\left(L_{n}\leq d_{n,\alpha }\right)\right\} \end{array}$$

Thus, we need to find $E\left\{\varphi_{1}\varphi_{1}^{T}\right\}$, $E\left\{\varphi_{3}\varphi_{1}^{T}I\left(L_{n}\leq d_{n,\alpha }\right)\right\}$ and $E\left\{\varphi_{3}\varphi_{3}^{T}I\left(L_{n}\leq d_{n,\alpha }\right)\right\}$, The first term is $E\left\{\varphi_{1}\varphi_{1}^{T}\right\}=B_{11.2}^{-1}+\mu_{11.2}\mu_{11.2}^{T}$. Using Lemma 1, the third term is computed as:

$$E\left\{\varphi_{3}\varphi_{3}^{T}I\left(L_{n}\leq d_{n,\alpha }\right)\right\}=\Phi H_{p_{2}+2}\left(\chi_{p_{2}}^{2};\Delta \right)+\delta \delta^{T}H_{p_{2}+4}\left(\chi_{p_{2}}^{2};\Delta \right).$$

The second term $E\left\{\varphi_{3}\varphi_{1}^{T}I\left(L_{n}\leq d_{n,\alpha }\right)\right\}$ can be computed from normal theory as

$$\begin{array}{cc} E\left\{\varphi_{3}\varphi_{1}^{T}I\left(L_{n}\leq d_{n,\alpha }\right)\right\} & =E\left\{E\left(\varphi_{3}\varphi_{1}^{T}I\left(L_{n}\leq d_{n,\alpha }\right)|\varphi_{3}\right)\right\}=E\left\{\varphi_{3}E\left(\varphi_{1}^{T}I\left(L_{n}\leq d_{n,\alpha }\right)|\varphi_{3}\right)\right\}\\ \; & =E\left\{\varphi_{3}{\left[-\mu_{11.2}+\left(\varphi_{3}-\delta \right)\right]}^{T}I\left(L_{n}\leq d_{n,\alpha }\right)\right\}\\ \; & =-E\left\{\varphi_{3}\mu_{11.2}I\left(L_{n}\leq d_{n,\alpha }\right)\right\}+E\left\{\varphi_{3}{\left(\varphi_{3}-\delta \right)}^{T}I\left(L_{n}\leq d_{n,\alpha }\right)\right\}\\ \; & =-\mu_{11.2}^{T}E\left\{\varphi_{3}I\left(L_{n}\leq d_{n,\alpha }\right)\right\}+E\left\{\varphi_{3}\varphi_{3}^{T}I\left(L_{n}\leq d_{n,\alpha }\right)\right\}\\ \; & -E\left\{\varphi_{3}\delta^{T}I\left(L_{n}\leq d_{n,\alpha }\right)\right\}\\ \; & =-\mu_{11.2}^{T}\delta H_{p_{2}+2}\left(\chi_{p_{2}}^{2};\Delta \right)+\{Cov\left(\varphi_{3}\varphi_{3}^{T}\right)H_{p_{2}+2}\left(\chi_{p_{2}}^{2};\Delta \right)\\ \; & +E\left(\varphi_{3}\right)E\left(\varphi_{3}^{T}\right)H_{p_{2}+4}\left(\chi_{p_{2}}^{2};\Delta \right)-\delta \delta^{T}H_{p_{2}+2}\left(\chi_{p_{2}}^{2};\Delta \right)\}\\ \; & =-\mu_{11.2}^{T}\delta H_{p_{2}+2}\left(\chi_{p_{2}}^{2};\Delta \right)+\Phi H_{p_{2}+2}\left(\chi_{p_{2}}^{2};\Delta \right)+\delta \delta^{T}H_{p_{2}+4}\left(\chi_{p_{2}}^{2};\Delta \right)\\ \; & -\delta \delta^{T}H_{p_{2}+2}\left(\chi_{p_{2}}^{2};\Delta \right) \end{array}$$

Putting all the terms together and simplifying, we obtain

$$\begin{array}{cc} \mathrm{Cov}({\hat{\beta }}_{1}^{\mathrm{RPT}}) & \\ = & \mu_{11.2}\mu_{11.2}^{T}+2\mu_{11.2}^{T}\delta H_{p_{2}+2}\left(\chi_{p_{2}}^{2};\Delta \right)+B_{11.2}^{-1}-\Phi H_{p_{2}+2}\left(\chi_{p_{2}}^{2};\Delta \right)-\delta \delta^{T}H_{p_{2}+4}\left(\chi_{p_{2}}^{2};\Delta \right)\\ + & 2\delta \delta^{T}H_{p_{2}+2}\left(\chi_{p_{2}}^{2};\Delta \right)\\ = & B_{11.2}^{-1}+\mu_{11.2}\mu_{11.2}^{T}+2\mu_{11.2}^{T}\delta H_{p_{2}+2}\left(\chi_{p_{2}}^{2};\Delta \right)-\Phi H_{p_{2}+2}\left(\chi_{p_{2}}^{2};\Delta \right)\\ & +\delta \delta^{T}\left[2H_{p_{2}+2}\left(\chi_{p_{2}}^{2};\Delta \right)-H_{p_{2}+4}\left(\chi_{p_{2}}^{2};\Delta \right)\right]. \end{array}$$

The asymptotic covariance of the estimator ${\hat{\beta }}_{1}^{\mathrm{RSE}}$ can be obtained as

$$\begin{array}{cc} \mathrm{Cov}({\hat{\beta }}_{1}^{\mathrm{RSE}}) & =E\{\lim_{n\to \infty }\sqrt{n}\left({\hat{\beta }}_{1}^{\mathrm{RSE}}-\beta_{1}\right)\sqrt{n}{\left({\hat{\beta }}_{1}^{\mathrm{RSE}}-\beta_{1}\right)}^{T}\}\\ \; & =E\{\lim_{n\to \infty }n[\left({\hat{\beta }}_{1}^{\mathrm{RFM}}-\beta_{1})-\left({\hat{\beta }}_{1}^{\mathrm{RFM}}-{\hat{\beta }}_{1}^{\mathrm{RSM}}\right)\left(p_{2}-2\right)L_{n}^{-1}\right]\\ \; & \left[{\left({\hat{\beta }}_{1}^{\mathrm{RFM}}-\beta_{1})-\left({\hat{\beta }}_{1}^{\mathrm{RFM}}-{\hat{\beta }}_{1}^{\mathrm{RSM}}\right)\left(p_{2}-2\right)L_{n}^{-1}\right]}^{T}\right\}\\ \; & =E\left\{\left[\varphi_{1}-\varphi_{3}\left(p_{2}-2\right)L_{n}^{-1}\right]{\left[\varphi_{1}-\varphi_{3}\left(p_{2}-2\right)L_{n}^{-1}\right]}^{T}\right\}\\ \; & =E\left\{\varphi_{1}\varphi_{1}^{T}-2\left(p_{2}-2\right)\varphi_{3}\varphi_{1}^{T}L_{n}^{-1}+{\left(p_{2}-2\right)}^{2}\varphi_{3}\varphi_{3}^{T}L_{n}^{-2}\right\} \end{array}$$

We need to compute $E\left\{\varphi_{3}\varphi_{3}^{T}L_{n}^{-2}\right\}$ and $E\left\{\varphi_{3}\varphi_{1}^{T}L_{n}^{-1}\right\}$. By using Lemma 1, the first term is obtained as follows:

$$E\left\{\varphi_{3}\varphi_{3}^{T}L_{n}^{-2}\right\}=\Phi E\left(\chi_{p_{2}+2}^{-4}\left(\Delta \right)\right)+\delta \delta^{T}E\left(\chi_{p_{2}+4}^{-4}\left(\Delta \right)\right).$$

The second term is computed from normal theory

$$\begin{array}{cc} E\left\{\varphi_{3}\varphi_{1}^{T}L_{n}^{-1}\right\} & =E\left\{E\left(\varphi_{3}\varphi_{1}^{T}L_{n}^{-1}|\varphi_{3}\right)\right\}=E\left\{\varphi_{3}E\left(\varphi_{1}^{T}L_{n}^{-1}|\varphi_{3}\right)\right\}\\ \; & =E\left\{\varphi_{3}{\left[-\mu_{11.2}+\left(\varphi_{3}-\delta \right)\right]}^{T}L_{n}^{-1}\right\}\\ \; & =-E\left\{\varphi_{3}\mu_{11.2}L_{n}^{-1}\right\}+E\left\{\varphi_{3}{\left(\varphi_{3}-\delta \right)}^{T}L_{n}^{-1}\right\}\\ \; & =-\mu_{11.2}^{T}E\left\{\varphi_{3}L_{n}^{-1}\right\}+E\left\{\varphi_{3}\varphi_{3}^{T}L_{n}^{-1}\right\}-E\left\{\varphi_{3}\delta^{T}L_{n}^{-1}\right\} \end{array}$$

From above, we can find $E\left\{\varphi_{3}\delta^{T}L_{n}^{-1}\right\}=\delta \delta^{T}E\left(\chi_{p_{2}+2}^{-2}\left(\Delta \right)\right)$ and $E\left\{\varphi_{3}L_{n}^{-1}\right\}=\delta E\left(\chi_{p_{2}+2}^{-2}\left(\Delta \right)\right)$. Putting these terms together and simplifying, we obtain

$$\begin{array}{cc} \mathrm{Cov}({\hat{\beta }}_{1}^{\mathrm{RSE}}) & =B_{11.2}^{-1}+\mu_{11.2}\mu_{11.2}^{T}+2\left(p_{2}-2\right)\mu_{11.2}^{T}\delta E\left(\chi_{p_{2}+2}^{-2}\left(\Delta \right)\right)\\ \; & -\left(p_{2}-2\right)\Phi \left\{2E\left(\chi_{p_{2}+2}^{-2}\left(\Delta \right)\right)-\left(p_{2}-2\right)E\left(\chi_{p_{2}+2}^{-4}\left(\Delta \right)\right)\right\}\\ + & \left(p_{2}-2\right)\delta \delta^{T}\left\{-2E\left(\chi_{p_{2}+4}^{-2}\left(\Delta \right)\right)+2E\left(\chi_{p_{2}+2}^{-2}\left(\Delta \right)\right)+\left(p_{2}-2\right)E\left(\chi_{p_{2}+4}^{-4}\left(\Delta \right)\right)\right\}. \end{array}$$

Since ${\hat{\beta }}_{1}^{\mathrm{RPS}}={\hat{\beta }}_{1}^{\mathrm{RSE}}-\left({\hat{\beta }}_{1}^{\mathrm{RFM}}-{\hat{\beta }}_{1}^{\mathrm{RSM}}\right)\left\{1-\left(p_{2}-2\right)L_{n}^{-1}\right\}I\left(L_{n}\leq p_{2}-2\right).$ We derive the covariance of the estimator ${\hat{\beta }}_{1}^{\mathrm{RPS}}$ as follows.

$$\begin{array}{cc} \mathrm{Cov}({\hat{\beta }}_{1}^{\mathrm{RPS}}) & =E\left\{\lim_{n\to \infty }\sqrt{n}\left({\hat{\beta }}_{1}^{\mathrm{RPS}}-\beta_{1}\right)\sqrt{n}{\left({\hat{\beta }}_{1}^{\mathrm{RPS}}-\beta_{1}\right)}^{T}\right\}\\ \; & =E\{\lim_{n\to \infty }\sqrt{n}\left({\hat{\beta }}_{1}^{\mathrm{RSE}}-\beta_{1}\right)-\sqrt{n}\left({\hat{\beta }}_{1}^{\mathrm{RFM}}-{\hat{\beta }}_{1}^{\mathrm{RSM}}\right)\left\{1-\left(p_{2}-2\right)L_{n}^{-1}\right\}I\left(L_{n}\leq p_{2}-2\right)\\ \; & \times {\left[\sqrt{n}\left({\hat{\beta }}_{1}^{\mathrm{RSE}}-\beta_{1}\right)-\sqrt{n}\left({\hat{\beta }}_{1}^{\mathrm{RFM}}-{\hat{\beta }}_{1}^{\mathrm{RSM}}\right)\left\{1-\left(p_{2}-2\right)L_{n}^{-1}\right\}I\left(L_{n}\leq p_{2}-2\right)\right]}^{T}\}\\ \; & =E\{\lim_{n\to \infty }\sqrt{n}\left({\hat{\beta }}_{1}^{\mathrm{RSE}}-\beta_{1}\right)\sqrt{n}{\left({\hat{\beta }}_{1}^{\mathrm{RSE}}-\beta_{1}\right)}^{T}-2\varphi_{3}\sqrt{n}{\left({\hat{\beta }}_{1}^{\mathrm{RSE}}-\beta_{1}\right)}^{T}\left\{1-\left(p_{2}-2\right)L_{n}^{-1}\right\}I\left(L_{n}\leq p_{2}-2\right)\\ \; & +\varphi_{3}\varphi_{3}^{T}{\left\{1-\left(p_{2}-2\right)L_{n}^{-1}\right\}}^{2}I\left(L_{n}\leq p_{2}-2\right)\}\\ \; & =\mathrm{Cov}\left({\hat{\beta }}_{1}^{\mathrm{RSE}}\right)-2E\left\{\lim_{n\to \infty }\varphi_{3}\sqrt{n}{\left({\hat{\beta }}_{1}^{\mathrm{RSE}}-\beta_{1}\right)}^{T}{\left\{1-\left(p_{2}-2\right)L_{n}^{-1}\right\}}^{2}I\left(L_{n}\leq p_{2}-2\right)\right\}\\ \; & +E\left\{\lim_{n\to \infty }\varphi_{3}\varphi_{3}^{T}{\left\{1-\left(p_{2}-2\right)L_{n}^{-1}\right\}}^{2}I\left(L_{n}\leq p_{2}-2\right)\right\}\\ \; & =\mathrm{Cov}\left({\hat{\beta }}_{1}^{\mathrm{RSE}}\right)-2E\left\{\lim_{n\to \infty }\varphi_{3}\varphi_{1}^{T}\left\{1-\left(p_{2}-2\right)L_{n}^{-1}\right\}I\left(L_{n}\leq p_{2}-2\right)\right\}\\ \; & +2E\left\{\lim_{n\to \infty }\varphi_{3}\varphi_{3}^{T}\left(p_{2}-2\right)L_{n}^{-1}\left\{1-\left(p_{2}-2\right)L_{n}^{-1}\right\}I\left(L_{n}\leq p_{2}-2\right)\right\}\\ \; & +E\left\{\lim_{n\to \infty }\varphi_{3}\varphi_{3}^{T}{\left\{1-\left(p_{2}-2\right)L_{n}^{-1}\right\}}^{2}I\left(L_{n}\leq p_{2}-2\right)\right\}\\ \; & =\mathrm{Cov}\left({\hat{\beta }}_{1}^{\mathrm{RSE}}\right)-2E\left\{\lim_{n\to \infty }\varphi_{3}\varphi_{1}^{T}\left\{1-\left(p_{2}-2\right)L_{n}^{-1}\right\}I\left(L_{n}\leq p_{2}-2\right)\right\}\\ \; & -E\left\{\lim_{n\to \infty }\varphi_{3}\varphi_{3}^{T}{\left(p_{2}-2\right)}^{2}L_{n}^{-2}I\left(L_{n}\leq p_{2}-2\right)\right\}+E\left\{\lim_{n\to \infty }\varphi_{3}\varphi_{3}^{T}I\left(L_{n}\leq p_{2}-2\right)\right\} \end{array}$$

We first compute the last term in the equation above $E\left\{\varphi_{3}\varphi_{3}^{T}I\left(L_{n}\leq p_{2}-2\right)\right\}$ as $E\left\{\varphi_{3}\varphi_{3}^{T}I\left(L_{n}\leq p_{2}-2\right)\right\}=\Phi H_{p_{2}+2}\left(p_{2}-2;\Delta \right)+\delta \delta^{T}H_{p_{2}+4}\left(p_{2}-2;\Delta \right).$ Using Lemma 1 and from the normal theory, we find,

$$\begin{array}{cc} \; & E\left\{\varphi_{3}\varphi_{1}^{T}\left\{1-\left(p_{2}-2\right)L_{n}^{-1}\right\}I\left(L_{n}\leq p_{2}-2\right)\right\}\\ \; & =E\left\{E\left(\varphi_{3}\varphi_{1}^{T}\left\{1-\left(p_{2}-2\right)L_{n}^{-1}\right\}I\left(L_{n}\leq p_{2}-2\right)|\varphi_{3}\right)\right\}\\ \; & =E\left\{\varphi_{3}E\left(\varphi_{1}^{T}\left\{1-\left(p_{2}-2\right)L_{n}^{-1}\right\}I\left(L_{n}\leq p_{2}-2\right)|\varphi_{3}\right)\right\}\\ \; & =E\left\{\varphi_{3}{\left[\mu_{11.2}+\left(\varphi_{3}-\delta \right)\right]}^{T}\left\{1-\left(p_{2}-2\right)L_{n}^{-1}\right\}I\left(L_{n}\leq p_{2}-2\right)\right\}\\ \; & =-\mu_{11.2}E\left(\varphi_{3}\left\{1-\left(p_{2}-2\right)L_{n}^{-1}\right\}I\left(L_{n}\leq p_{2}-2\right)\right)\\ \; & +E\left(\varphi_{3}\varphi_{3}^{T}\left\{1-\left(p_{2}-2\right)L_{n}^{-1}\right\}I\left(L_{n}\leq p_{2}-2\right)\right)\\ \; & -E\left(\varphi_{3}\delta^{T}\left\{1-\left(p_{2}-2\right)L_{n}^{-1}\right\}I\left(L_{n}\leq p_{2}-2\right)\right)\\ \; & =-\delta \mu_{11.2}^{T}E\left(\left\{1-\left(p_{2}-2\right)\chi_{p_{2}+2}^{-2}\left(\Delta \right)\right\}I\left(\chi_{p_{2}+2}^{-2}\left(\Delta \right)\leq p_{2}-2\right)\right)\\ \; & +\Phi E\left(\left\{1-\left(p_{2}-2\right)\chi_{p_{2}+2}^{-2}\left(\Delta \right)\right\}I\left(\chi_{p_{2}+2}^{-2}\left(\Delta \right)\leq p_{2}-2\right)\right)\\ \; & +\delta \delta^{T}E\left(\left\{1-\left(p_{2}-2\right)\chi_{p_{2}+4}^{-2}\left(\Delta \right)\right\}I\left(\chi_{p_{2}+4}^{-2}\left(\Delta \right)\leq p_{2}-2\right)\right)\\ \; & -\delta \delta^{T}E\left(\left\{1-\left(p_{2}-2\right)\chi_{p_{2}+4}^{-2}\left(\Delta \right)\right\}I\left(\chi_{p_{2}+4}^{-2}\left(\Delta \right)\leq p_{2}-2\right)\right). \end{array}$$

$$\begin{array}{cc} E\left\{\varphi_{3}\varphi_{3}^{T}{\left(p_{2}-2\right)}^{2}L_{n}^{-2}I\left(L_{n}\leq p_{2}-2\right)\right\} & ={\left(p_{2}-2\right)}^{2}\Phi E\left(\chi_{p_{2}+2}^{-4}\left(\Delta \right)I\left(\chi_{p_{2}+2}^{2}\left(\Delta \right)\leq p_{2}-2\right)\right)\\ \; & +{\left(p_{2}-2\right)}^{2}\delta \delta^{T}E\left(\chi_{p_{2}+2}^{-4}\left(\Delta \right)I\left(\chi_{p_{2}+2}^{2}\left(\Delta \right)\leq p_{2}-2\right)\right) \end{array}$$

Putting all the terms together, we obtain

$$\begin{array}{cc} \mathrm{Cov}({\hat{\beta }}_{1}^{\mathrm{RPS}}) & =\mathrm{Cov}\left({\hat{\beta }}_{1}^{\mathrm{RSE}}\right)+2\delta \mu_{11.2}^{T}E\left(\left\{1-\left(p_{2}-2\right)\chi_{p_{2}+2}^{-2}\left(\Delta \right)\right\}I\left(\chi_{p_{2}+2}^{2}\left(\Delta \right)\leq p_{2}-2\right)\right)\\ \; & -2\Phi E\left(\left\{1-\left(p_{2}-2\right)\chi_{p_{2}+2}^{-2}\left(\Delta \right)\right\}I\left(\chi_{p_{2}+2}^{2}\left(\Delta \right)\leq p_{2}-2\right)\right)\\ \; & -2\delta \delta^{T}E\left(\left\{1-\left(p_{2}-2\right)\chi_{p_{2}+4}^{-2}\left(\Delta \right)\right\}I\left(\chi_{p_{2}+4}^{2}\left(\Delta \right)\leq p_{2}-2\right)\right)\\ \; & +2\delta \delta^{T}E\left(\left\{1-\left(p_{2}-2\right)\chi_{p_{2}+2}^{-2}\left(\Delta \right)\right\}I\left(\chi_{p_{2}+2}^{2}\left(\Delta \right)\leq p_{2}-2\right)\right)\\ \; & -{\left(p_{2}-2\right)}^{2}\Phi E\left(\chi_{p_{2}+2}^{-4}\left(\Delta \right)I\left(\chi_{p_{2}+2,\alpha }^{2}\left(\Delta \right)\leq p_{2}-2\right)\right)\\ \; & -{\left(p_{2}-2\right)}^{2}\delta \delta^{T}E\left(\chi_{p_{2}+2}^{-4}\left(\Delta \right)I\left(\chi_{p_{2}+2}^{2}\left(\Delta \right)\leq p_{2}-2\right)\right)\\ \; & +\Phi H_{p_{2}+2}\left(p_{2}-2;\Delta \right)+\delta \delta^{T}H_{p_{2}+4}\left(p_{2}-2;\Delta \right). \end{array}$$

 □

## References

[1] Laird N.M., Ware J.H. (1982). Random-effects models for longitudinal data. Biometrics.

[2] Longford N. (1993). Regression analysis of multilevel data with measurement error. Br. J. Math. Stat. Psychol..

[3] Tibshirani R. (1996). Regression shrinkage and selection via the lasso. J. R. Stat. Soc. Ser. B (Methodol.).

[4] Zou H. (2006). The adaptive lasso and its oracle properties. J. Am. Stat. Assoc..

[5] Tran M.N. (2011). The loss rank criterion for variable selection in linear regression analysis. Scand. J. Stat..

[6] Huang J., Ma S., Zhang C.H. (2008). Adaptive Lasso for sparse high-dimensional regression models. Stat. Sin..

[7] Kim Y., Choi H., Oh H.S. (2008). Smoothly clipped absolute deviation on high dimensions. J. Am. Stat. Assoc..

[8] Wang H., Leng C. (2007). Unified LASSO estimation by least squares approximation. J. Am. Stat. Assoc..

[9] Yuan M., Lin Y. (2006). Model selection and estimation in regression with grouped variables. J. R. Stat. Soc. Ser. B (Stat. Methodol..

[10] Leng C., Lin Y., Wahba G. (2006). A note on the lasso and related procedures in model selection. Stat. Sin..

[11] Park T., Casella G. (2008). The bayesian lasso. J. Am. Stat. Assoc..

[12] Greenlaw K., Szefer E., Graham J., Lesperance M., Nathoo F.S., Initiative A.D.N. (2017). A Bayesian group sparse multi-task regression model for imaging genetics. Bioinformatics.

[13] Ahmed S.E., Nicol C.J. (2012). An application of shrinkage estimation to the nonlinear regression model. Comput. Stat. Data Anal..

[14] Ahmed S.E., Raheem S.E. (2012). Shrinkage and absolute penalty estimation in linear regression models. Wiley Interdiscip. Rev. Comput. Stat..

[15] Lisawadi S., Kashif Ali Shah M., Ejaz Ahmed S. (2016). Model selection and post estimation based on a pretest for logistic regression models. J. Stat. Comput. Simul..

[16] Ahmed S.E., Opoku E.A. (2017). Submodel selection and post-estimation of the linear mixed models. Proceedings of the Tenth International Conference on Management Science and Engineering Management.

[17] Raheem S.E., Ahmed S.E., Doksum K.A. (2012). Absolute penalty and shrinkage estimation in partially linear models. Comput. Stat. Data Anal..

[18] Geladi P., Kowalski B.R. (1986). Partial least-squares regression: A tutorial. Anal. Chim. Acta.

[19] Liu K. (2003). Using Liu-type estimator to combat collinearity. Commun. Stat.-Theory Methods.

[20] Hoerl A.E., Kennard R.W. (1970). Ridge regression: Biased estimation for nonorthogonal problems. Technometrics.

[21] Yüzbaşı B., Ejaz Ahmed S. (2016). Shrinkage and penalized estimation in semi-parametric models with multicollinear data. J. Stat. Comput. Simul..

[22] Yüzbası B., Ahmed S.E., Güngör M. (2017). Improved penalty strategies in linear regression models. REVSTAT J..

[23] Knight K., Fu W. (2000). Asymptotics for lasso-type estimators. Ann. Stat..

[24] Belsley D.A. (1991). Conditioning Diagnostics: Collinearity and Weak Data in Regression.

[25] Twisk J., Kemper H., Mellenbergh G. (1995). Longitudinal development of lipoprotein levels in males and females aged 12–28 years: The Amsterdam Growth and Health Study. Int. J. Epidemiol..

[26] Nie Y., Opoku E., Yasmin L., Song Y., Wang J., Wu S., Scarapicchia V., Gawryluk J., Wang L., Cao J. (2020). Spectral dynamic causal modelling of resting-state fMRI: An exploratory study relating effective brain connectivity in the default mode network to genetics. Stat. Appl. Genet. Mol. Biol..

[27] Ahmed S.E., Kim H., Yıldırım G., Yüzbaşı B. (2016). High-Dimensional Regression Under Correlated Design: An Extensive Simulation Study. International Workshop on Matrices and Statistics.

[28] Ejaz Ahmed S., Yüzbaşı B. (2016). Big data analytics: Integrating penalty strategies. Int. J. Manag. Sci. Eng. Manag..

[29] Ahmed S.E., Yüzbaşı B. (2017). High dimensional data analysis: Integrating submodels. Big and Complex Data Analysis.

[30] Judge G.G., Bock M.E. (1978). The Statistical Implication of Pre-Test and Steinrule Estimators in Econometrics.

